# IRF1 mediates airway epithelial innate memory through priming BRD4- EP300 activity

**DOI:** 10.3389/fimmu.2026.1923234

**Published:** 2026-07-28

**Authors:** Xiaofang Xu, Robert L. Gearhart, Monica Yun Liu, Allan R. Brasier

**Affiliations:** 1 Department of Medicine, University of Wisconsin-Madison School of Medicine and Public Health (SMPH), Madison, WI, United States; 2 Division of Allergy, Pulmonary, and Critical Care, Department of Medicine, University of Wisconsin-Madison, Madison, WI, United States; 3 Institute for Clinical and Translational Research, University of Wisconsin-Madison, Madison, WI, United States

**Keywords:** H3K27 acetylation, innate immune memory, innate immune response, innate training, TLR3

## Abstract

**Introduction:**

Sentinel cells of the airway epithelium are repetitively exposed to RNA virus infections. Here, the production of pathogen-associated molecular patterns (PAMPs) activates the innate immune response (IIR), inducing antiviral defenses, activating adaptive immunity and stimulating epithelial repair. The question whether repeated exposures produce innate “memory”, where heightened inflammatory and injury/repair responses are produced upon subsequent exposure, is unknown.

**Methods:**

We establish a reproducible model of innate memory in tumor protein (TP63)+/keratin (KRT)5+ human small airway basal epithelial cells by repetitive activation of the Toll like receptor (TLR3) pathway (a.k.a. “training”). Mechanisms of IRF1-dependent target gene remodeling are investigated by CRISPR/Cas9 knockdown, chromatin immunoprecipitation, co-immunoprecipitation and through use of selective inhibitors of the BRD4 chromatin remodeling complex (CRC).

**Results:**

We observe “training” increases basal expression of a network of intrinsic IIR genes, reducing Respiratory Syncytial Virus (RSV) replication upon subsequent exposure. In addition, trained hSAECs enter an epithelial mesenchymal plasticity (EMP) program, characterized by cell shape changes, increased expression of cytoplasmic vimentin and core mesenchymal regulators. Mechanistically, we find that Interferon Regulatory Factor 1 (IRF1) is required for innate memory because IRF1-deficient cells fail to activate basal intrinsic IIR. In TLR3 activation, IRF1 is incorporated into the bromodomain containing protein 4 (BRD4) chromatin remodeling complex (CRC), a complex containing the histone H3 acetyltransferase, EP300/p300. In acute TLR3 activation, the binding of BRD4 is increased on intrinsic IIR promoters, yet this binding is unaffected by training. Instead, we find training induces EP300 binding and increases H3K27 acetylation (H3K27ac) at these regions. Small molecule inhibitors show that BRD4 is required for establishing innate memory and H3K27ac accumulation. Finally, we find that training enhances BRD4-EP300 interactions and that memory-induced H3K27ac formation is EP300-dependent.

**Conclusions:**

We conclude that hSAECs acquire innate memory through IRF1 coupling with the BRD4-EP300 CRC to activate intrinsic IIR and cell-state transition genes. Our data suggest that innate training is the consequence of dynamic BRD4 CRC interactions through enhanced EP300 recruitment that primes transcriptional responsiveness.

## 1 Introduction

The airway epithelium is repetitively exposed to allergens, environmental oxidants and viral pathogens throughout life. Airway epithelial injury is a component of common obstructive lung diseases including asthma and chronic obstructive lung disease producing worldwide morbidity (1). Specialized epithelial cells detect the presence of exogenous airway triggers activating innate immune responses (IIR) and cellular repair programs to restore homeostasis and restrict the spread of infection. Acutely, the IIR is protective; however, chronic or repetitive exposures lead to dysregulated injury repair responses, dysfunctional epithelial differentiation/goblet cell hyperplasia and barrier disruption (2–4). Understanding the molecular mechanisms how the airway epithelium responds to repetitive airway insults is an unmet knowledge gap.

In the pseudostratified conducting airway epithelium, basal cells play an important sentinel function through the expression of pattern recognition receptors (PRRs) stimulating secretion of IFNs and serving as sites for alarmin checkpoint in viral injury (5, 6). These cells constitute ~30% of the epithelial population and, importantly, express a spectrum of PRRs including the membrane and endosomal Toll-like receptor (TLR) 3, 7 and 8 as well as cytoplasmic RIG-I (DDX58) and MDA5 (IFIH1) (7, 8). Amongst these, TLR3 occupies a particularly important role in antiviral surveillance, recognizing double-stranded RNA (dsRNA), where its activation initiates expression of the mucosal type I and -III interferons (IFNs) (9). Consequently, basal cells produce high levels of IFN, and deficiencies in IFN secretion are associated with enhanced viral susceptibility (10). Although the mechanism for their acute anti-viral responses in amplification of IFN stimulated genes are extensively studied (11), the effects of chronic TLR3 activation are not well understood.

Seasonal and pandemic RNA virus infections are major causes of respiratory tract infections and exacerbations of chronic airway disease. In particular, the orthopneumovirus, Respiratory Syncytial Virus (RSV), is a major human pathogen responsible for 3.6 M hospitalizations and ~100,000 deaths in children under 5 years of age (12, 13). Because RSV does not confer long-term protective immunity, re-infection occurs throughout life. Approximately~40% of children have experienced at least two RSV infections by age two (13, 14), and RSV is a recognized cause of nosocomial pneumonia outbreaks. Clinically, the severity of RSV infection is reduced upon recurrent infection (15), despite the finding that RSV evades durable adaptive immunity.

In natural infections in both human and animal models, RSV readily disseminates into the lower respiratory tract where specialized sentinel epithelial cells secrete neutrophilic and Th2 polarizing chemokines, linked to atopy and asthma (16–18). To more precisely identify these sentinels, lineage tracing experiments have identified a population of multipotent cells within the small airways expressing Tumor protein 63 (TP63)+/Keratin 5 (KRT5)+ markers repopulate the epithelium after injury (19–21). Viruses trophic to the lower airway epithelium, including RSV, induce cell sloughing (22), a process that stimulates the basal cells to bypass regulatory checkpoints (6), to enter epithelial mesenchymal plasticity (EMP) to repopulate the disrupted epithelial barrier (19–21, 23). Basal progenitor epithelial cells are relatively resistant to RSV infection by virtue of the intrinsic innate immune response (IIR) (24). The intrinsic IIR is mediated by IFN-independent expression of a subgroup of IFN-stimulated genes regulated by the transcription factor interferon regulatory factor (IRF1). This intrinsic IIR includes the signature genes Bone Marrow Stromal Cell Antigen 2 (BST2), Interferon-Induced Transmembrane Protein 1 (IFITM1), and 2`-5`-Oligoadenylate Synthetase 2 (OAS2), whose basal expression provide broad antiviral protection (24, 25). Activation of the intrinsic IIR is facilitated by the BRD4 chromatin remodeling complex (CRC) enabling progenitor cells to rapidly mount an innate host response (24, 26).

To investigate adaptive mechanisms to repetitive viral PAMP exposure, we find that repetitive TLR3 activation promotes innate memory in Trp63^+^/Krt5^+^ basal cells, producing elevated *BST2/IFITM1/OAS2* expression and substantially reducing RSV transcription upon challenge. IRF1 mediates memory state of the intrinsic IIR genes as well as the coupled entry into EMP. We find IRF1 is incorporated into the BRD4 CRC containing the EP300 histone acetyltransferase producing exaggerated H3K27ac binding to intrinsic IIR promoters, “priming” their response. Finally, we find that training enhances BRD4-EP300 interactions and that memory-induced H3K27ac formation is EP300-dependent. These findings have important implications for understanding strategies for modulating innate immunity in developing viral prophylaxis strategies and enhancing tissue repair from viral infections.

## 2 Materials and methods

### 2.1 Cell cultures and innate training

TP63^+^/KRT5+ human small airway epithelial (hSAECs) were grown in small airway epithelial cell growth medium (SAGM, Lonza, Walkersville, MD, USA) in 5% CO_2_. IRF1 knockdown cells were generated using lentiCRISPRv2 and sgRNA target sequence 5’-CACCTCCTCGATATCTGGCA-3’ targeting exon 4 as previously described (24). IRF1 KD cells were maintained in SAGM with 10 µg/ml puromycin.

Primary airway organoids were isolated from distal lung tissue (obtained under IRB-approved protocol #2023-1729) from a deceased donor (organ donor whose lungs could not be used for transplant). After expansion, basal cells were enriched by FACS sorting for NGFR^+^ (27). After FACS, cells were dissociated and stimulated with 1 µg/ml poly(I:C).

Sucrose cushion-purified RSV Long strain was prepared using established methods (28). For RSV infection, cells were mock or RSV-infected at a multiplicity of infection (MOI) = 1.0 and left in the medium until harvest. An MOI of 1.0 was selected to synchronize infection of the culture.

For innate training, hSAECs were stimulated with the TLR3 agonist, polyinosinic–polycytidylic acid (poly(I:C), Millipore Sigma) at 50 μg/ml final concentration for 4 h daily for 3 d. After each stimulation, the cells were washed and cultured in SAGM medium. This dose was determined empirically to maximize IRF1 activation without inducing cell death. For BRD4 inhibition experiments, ZL0969 was synthesized and HPLC-purified (29). ZL0969 was used at 10 μM concentration in cell culture medium added at the time of poly(I:C) stimulation.

### 2.2 Quantitative real time-PCR

Total cellular RNA was extracted with RNeasy kit (Qiagen 75144). cDNA were synthesized with LunaScript RT supermix kit (NEB E3010) and amplified with NEBNext high-fidelity 2x PCR master mix (NEB M0541). Quantification of relative changes in gene expression was calculated using the ΔΔCt method and normalized to internal control peptidylprolyl isomerase A (PPIA). Primer sequences have been previously reported (24).

Strand-specific PCR detection of RSV was performed as previously described (30). Briefly, for strand-specific cDNA synthesis, reverse transcription reactions were prepared using the primer-free LunaScript RT Master Mix (NEB E3025) together with strand-specific RT primers, including positive- and negative-sense-specific primers. Quantitative PCR was subsequently performed using TaqMan Fast Advanced Master Mix (Applied Biosystems, 4444556), a strand-specific forward primer (positive-sense or negative-sense), a tag primer, and a TaqMan probe.

### 2.3 Co-immunoprecipitation and Western blot

Cells were trypsinized, pelleted and washed twice with cold phosphate-buffered saline (PBS). Cell pellets were then lysed in 1ml cold low ionic strength buffer (50 mM NaCl, 1% IGEPAL, 10% Glycerol, 10 mM HEPES, pH7.4) with fresh added proteinase inhibitor cocktail (1:100 vol/vol dilution, Sigma P8340), 1 mM DTT and 1 mM PMSF (Sigma P7626). The cell lysates were sonicated twice and centrifuged at 10,000xg for 10 minutes at 4 °C. For each sample, 100 μl of supernatants were reserved as input and separate western blot analysis. The remaining 450 microliter- uL of supernatant per group was incubated with 1.34 microliter- uL of IgG antibody (LS Bio, LS-C149375, 5 mg/ml) or 9ul of BRD4 antibody, 749 μg/ml). On the following day, 25 μL of protein G Dynabeads (Invitrogen 10004-D) was added to each sample and incubated for 2 hours at 4 °C on a rotator. Beads were then washed and samples were denatured in SDS sample buffer, heated at 95 °C for 5 min, and fractionated using Criterion TGX 4-15% PAGE gels. Proteins were transferred to PVDF membranes and probed with anti-IRF1 (Proteintech 11335-1-AP, 1:500), anti-phosphorylated Ser2 CTD RNA Pol II Ab (Abcam ab5095, 1:700), anti-p300 (Cell Signaling, 1:1000), anti-CBP (Cell Signaling 73895, 1:1000) or BRD4 (Cell Signaling 13440, 1:1000). Secondary detection was using VeriBlot (Abcam, ab131366, 1:1000) and Supersignal West Femto (Thermo, 34095) for co-IP western and HRP anti-rabbit IgG (Thermo P-10994, 1: 100000) for input western. Blots were imaged and bands quantified.

### 2.4 Immunofluorescence microscopy

hSAECs were plated on coverslips, and infected or treated as indicated. Afterwards, cells were fixed with 4% paraformaldehyde, permeabilized with 0.1% Triton X-100, blocked with 5% goat serum and incubated with primary Ab overnight (VIM: Sigma, V6630). On the second day, coverslips were washed with 0.2% Tween-20 and incubated with Alexa fluor goat secondary Ab. After one hour, cells were washed and mounted using ProLong Diamond Antifade Mountant with 4′,6-diamidino-2-phenylindole (DAPI, Thermo Fisher P36931). The cells were visualized in an ECHO Revolve fluorescence microscope.

### 2.5 Two-step chromatin IP -quantitative genomic PCR

XChIP was performed using sequential disuccinimidyl glutarate (DSG; 2 mM, 45 min at 22 °C, Thermo 20593) and methanol-free formadehyde (1% vol/vol in PBS, 10 min) crosslinking (53). The following antibodies were used: IRF1 (Proteintech 11335-1-AP), BRD4 (Cell Signaling 13440), p-Ser2 Pol II (Abcam ab5095); IgG was a nonspecific binding control (LS Bio LS-C149375). IPs were collected with 20 μl of Protein G coupled Dynabeads, washed, eluted and de-crosslinked. Gene enrichment of the de-crosslinked DNA was quantitated using promoter-specific primers (24). For each gene, enrichment was determined by normalizing to input DNA and calculating the fold change relative to the signal in unstimulated cells (31).

### 2.6 Proximity ligation assay

PLA was performed as previously described (32). Cells were treated, fixed with 4% paraformaldehyde, permeabilized with 0.1% Triton X-100, and incubated overnight with indicated pairwise combinations of rabbit BRD4 (Cell signaling 13440, 1:500) and mouse EP300. The nuclei were counterstained with DAPI, and the PLA signals were visualized with an ECHO fluorescent microscope at x 20 magnification.

### 2.7 Informatics and statistical analyses

Publicly available BRD4 (GEO GSE170772) and EP300 ChIP-Seq data sets (ENCODE ENCSR571KWZ) from unstimulated type II-like A549 cells were downloaded from GEO. ChIP seq summits from narrowbed format were used for signal quantification. Signal matrices were constructed with the Enriched Heatmap package in R, extending ± 1 Kb upstream and downstream of the peak. Rows were partitioned into promoter and enhancer groups on the basis of H3K4me1 and H3K27ac distributions and ranked by BRD4 signal intensity. EP300 peaks from the same locations were extracted and centered.

Statistical analyses were performed with Graph Pad Prism 9 (GraphPad Software, San Diego, CA). Results are expressed as mean ± SD. Normality and equal variance tests were performed to determine appropriate application of parametric statistical analyses. For multiple group experiments, one-way ANOVA was used with *post-hoc* Sidak T-tests for group-wise comparison between treatments. P values < 0.05 were considered to be statistically significant.

## 3 Results

### 3.1 TLR3 training constrains viral replication

**Figure 1:**
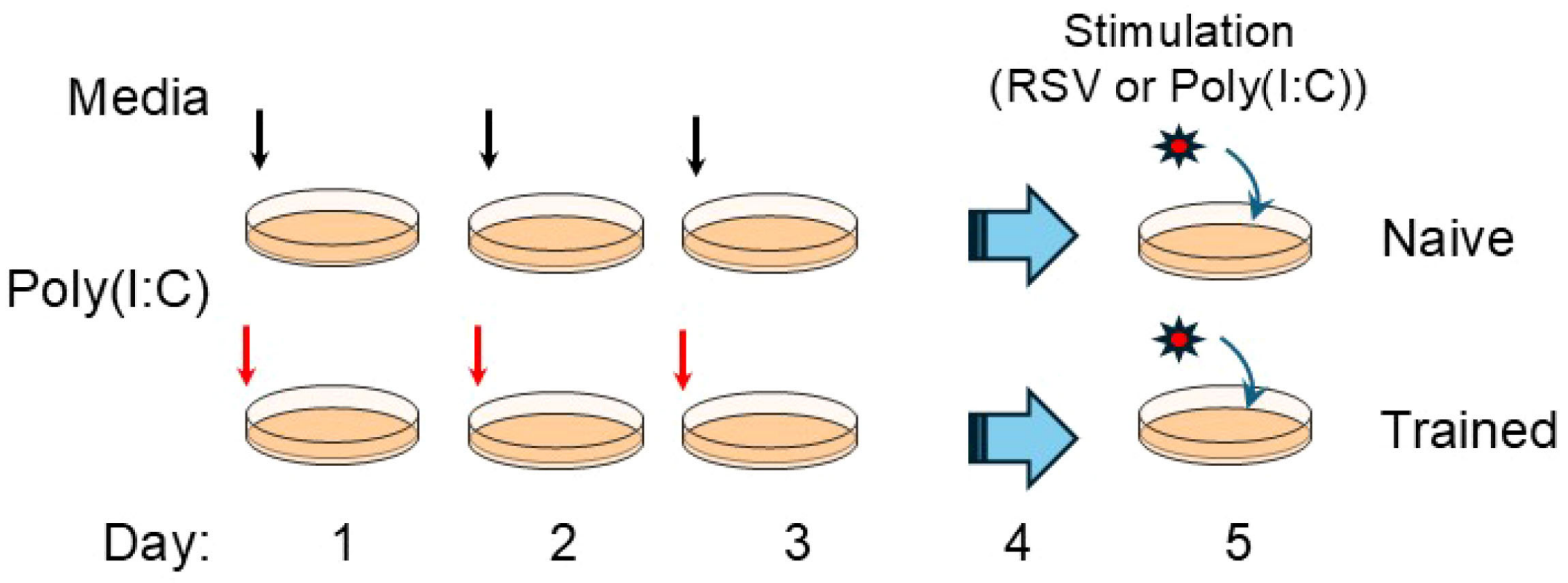
Innate training. Schematic diagram of innate training with daily mock treatment or poly(I:C) stimulation (4 h daily, 3 d). hSAECs maintained in submerged culture were split on day 4 to ensure equal cell density for subsequent treatments. On day 5, cells were challenged with either sucrose (mock), infected with purified RSV (MOI 1.0, 16 h) or stimulated with poly(I:C).

TLR3 plays a central role in the intrinsic IIR as the only TLR that mediates Type-I and III mucosal IFN production (9) by virtue of its unique coupling to the TRIF coactivator. Consequently TLR3-TRIF activates IRF1 through an autoregulatory pathway (33, 34). To explore the role of TLR3 training in basal cells, we conducted repetitive stimulation with the TLR3 agonist, poly(I:C), in submerged cell cultures of TP63^+^/KRT5^+^ small airway basal cells (hSAECs; **Figure 1**). hSAECs are lineage-restricted airway epithelial cells that self-replicate, activating metabolic reprogramming and extracellular matrix remodeling characteristic of basal cells *in vivo*, and differentiate into specialized ciliated, club and mucus-secreting goblet cells in air liquid interfaces, making these cells a valid airway basal cell model (24, 35). We noted that TLR3-stimulated (“trained’) hSAECs assumed a more elongated shape and lost cell boundaries formed upon confluency compared to naïve controls (not shown).

**Figure 2:**
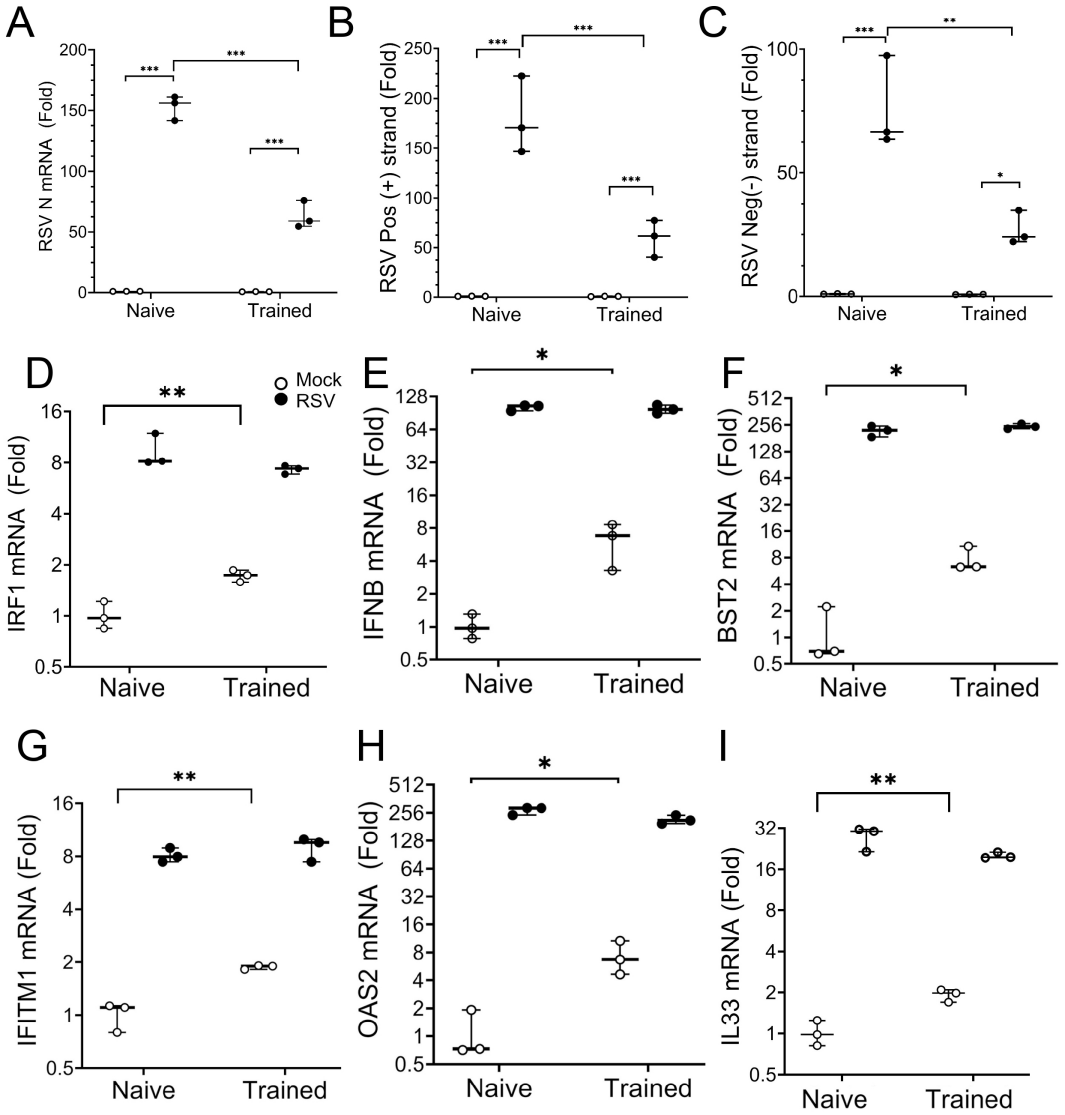
Innate training reduces RSV transcription. Strand specific analysis of RSV transcription and replication was assayed and expressed as fold change 2h post infection vs 16 h. **(A)** RSV N mRNA; **(B)**, RSV (+) strand replicative RNA; **(C)** RSV (–) strand replicative RNA. Each symbol is an independent replicate. **(D–H)** Q-RT-PCR of IIR genes. Shown is fold change mRNA normalized to PPIA as internal control. **(D)** *IRF1* mRNA; **(E)** *BST2* mRNA; **(F)** *IFITM1* mRNA; **(G)** *OAS2* mRNA; **(H)**, *IL33* mRNA. *P<0.05; **P< 0.01 *post-hoc*.

To explore the effect of training on the anti-viral response, we challenged naive and trained cells with sucrose-cushion purified RSV (MOI 1.0, 16h). We observed that trained cells supported less RSV transcription, producing 24% less RSV nucleoprotein (N) mRNA relative to that of naive hSAECs (150 ± 10-fold in naive cells vs 63 ± 11-fold in trained cells, P<0.05, n=3; **Figure 2A**). To better understand the effect on RSV transcription, we applied an established, strand-specific Q-RT-PCR assay, capable of detecting the presence of replicative intermediate (RI) RSV genomic RNA produced during viral replication (30). Here, positive-sense RNA is detected using strand-selective cDNA synthesis. We found that RSV (+) sense RI genomic RNA was reduced from 180 ± 39-fold in naive cells to 59 ± 18-fold in trained cells (P<0.001, n=3; **Figure 2B**). A similar reduction on the abundance of the RSV negative sense strand was produced (P<0.001, n=3; **Figure 2C**). These data indicate that TLR3 training produced a cell state that constrains viral replication.

### 3.2 TLR3 training activates the unstimulated tone of the intrinsic IIR

To understand this anti-viral phenomenon better, we examined the effect of training on the anti-viral intrinsic arm of the IIR. We observed that in mock-RSV naive hSAECs, IRF1 mRNA increased to 9.4 ± 2.2-fold in response to RSV infection relative to mock-infected controls, consistent with earlier observations (P<0.01, n=3; **Figure 2D**). By contrast, we observed that the level of *IRF1* mRNA in mock-infected trained hSAECs was increased by 1.7 ± 0.14-fold relative to that of naive hSAECs (P<0.01, n=3; **Figure 2D**), whereas induced *IRF1* mRNA after RSV infection was not significantly different from that of naïve cells (n.s.). Similarly, *IFNß* expression was activated 6.2 ± 2.7-fold in uninfected trained cells relative to that of naïve cells (P< 0.01, n=3; **Figure 2E**) yet the level of RSV induced *IFNß* mRNA was also similar in the trained vs naïve cells (n.s., n=3; **Figure 2E**). These findings suggested to us that the major effect of training is on tonic expression of the intrinsic IIR in the absence of infection.

To confirm that the IRF1 tone was enhanced in hSAECs after training, we examined the effect of repetitive TLR3 activation on basal expression of direct IRF1 targets. For this purpose, Bone Marrow Stromal Cell Antigen (BST2), Interferon Induced Transmembrane Protein 1 (IFITM1) and 2’-5’-Oligoadenylate Synthetase 2 (OAS2) were selected. BST2 physically tethers budding virions to the cell surface, whereas IFITM proteins prevent early viral fusion to cell membranes, and OAS2 is a dsRNA viral sensor and activator of RNase L to cleave RNA virus. Most importantly, these genes were selected as endogenous sensors of IRF1 tone because our previous work showed that: 1., IRF1 directly binds to their proximal promoters in chromatin immunoprecipitation; and 2., their expression is rate-limited by ambient IRF1 expression – not only does IRF1 silencing reduce their expression, ectopic IRF1 further transactivates their expression (24).

We observed that innate training increased *BST2* mRNA by 7.8 ± 2.6-fold relative to naive hSAECs (P<0.01, n=3, **Figure 2F**), *IFITM1* mRNA was increased by 1.9 ± 0.05-fold (P<0.001, n=3, **Figure 2G**) and *OAS2* mRNA was increased by 6 ± 1.9-fold (P<0.05, n=3, **Figure 2H**). As an analysis of checkpoint transition, *IL33* mRNA was similarly upregulated (P<0.05, n=3, **Figure 2I**). Collectively, these data indicate that TLR3 training enhanced the tonic expression of the intrinsic IIR to a level sufficient to reduce RSV transcription.

Because the trained hSAECs reduce the level of RSV transcription, study of inducible IIR in infected cells is potentially confounded. Lower levels of cytoplasmic RSV structural transcripts expressed in the trained cells reduce intracellular signaling through the cytoplasmic RNA helicases, confounding the assessment of training on responsiveness of the host IIR. Consequently, in this and future experiments, we stimulated hSAECs with poly(I:C) to better examine the effect of training on TLR3 pathway.

### 3.3 TLR3 training induces basal IIR in primary human basal cells

**Figure 3:**
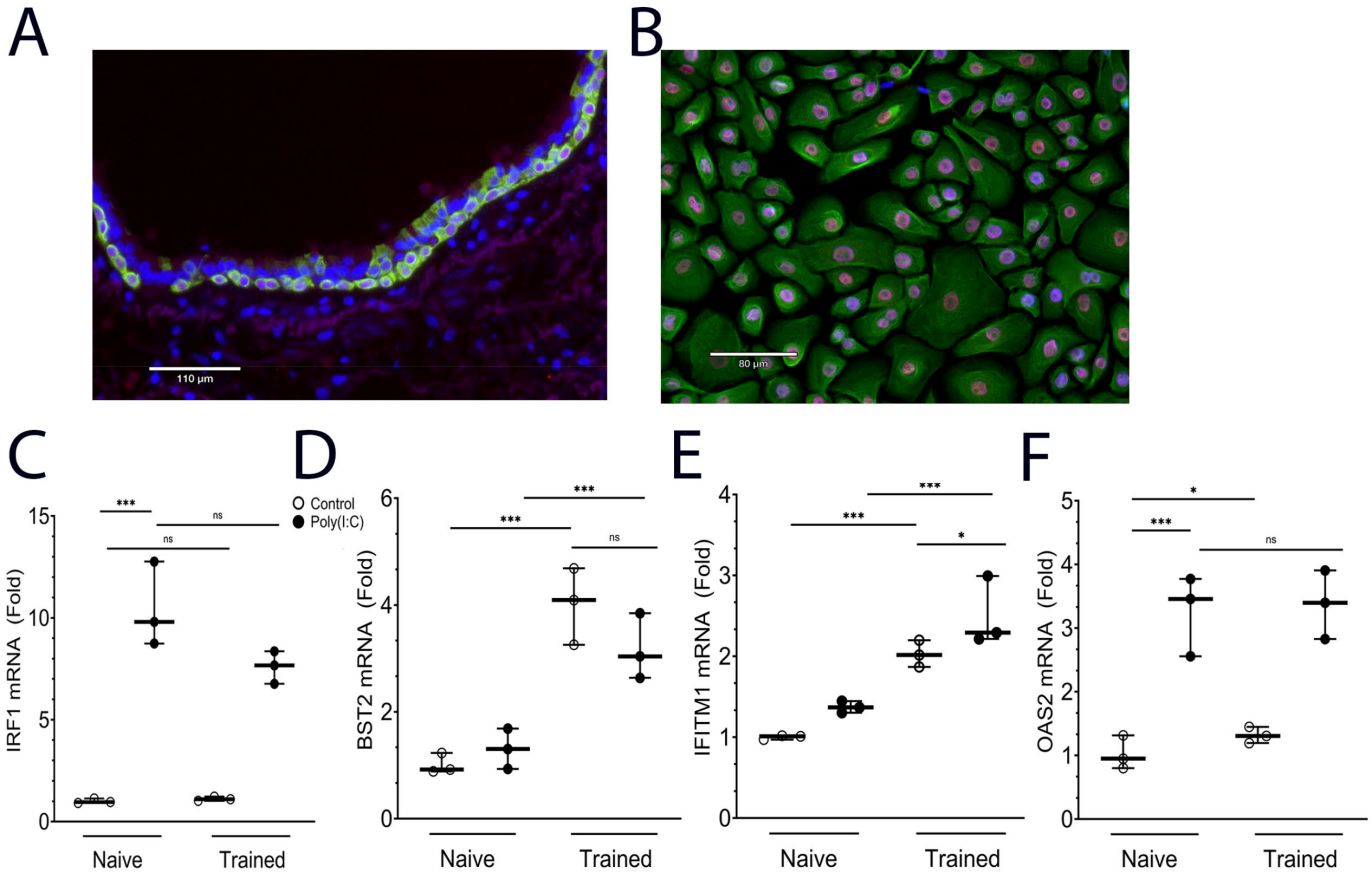
Training response in human Trp63^+^/Krt5^+^ basal cells. **(A)** IFM of human small bronchiole. Section was stained with anti-TP63 (red), Krt5+ (green) and nuclei stained with DAPI (blue). 10X magnification is shown. Note the uniform layer of basal cells underneath differentiated cells. Scale bar in microns. **(B)** Basal cell cultures dissociated from organoids and plated. Section was stained with anti-TP63 (red), KRT5+ (green) and nuclei stained with DAPI (blue). Scale bar in microns. Note homogenous expression of TP63 and KRT5 **(C–E)** Q-RT-PCR of fold change mRNA normalized to *PPIA* as internal control. **(C)** *IRF1* mRNA; **(D)** *BST2* mRNA; **(E)** *IFITM1* mRNA. **(F)** *OAS2* mRNA. N.s., not significant; *P<0.05; ***P< 0.001 *post-hoc*.

To confirm and extend this memory phenomenon, we explored the effects of training on primary human basal cells. In the human small airways TP63^+^/Krt5^+^ basal cells are a prominent population underlying ciliated cells adherent to the basal lamina, as seen by the immunofluorescence microscopy (IFM), **Figure 3A**. Airway organoids isolated from distal lung tissue were expanded and FACS sorted for NGFR expression. Basal cells were then plated, where they maintained TP63^+^/Krt5^+^ staining (36) (**Figure 3B**). These organoid-derived basal cells were then subjected to a modified training protocol (**Figure 1**), where cells were exposed to 1 µg/ml poly(I:C) to maintain viability. In these primary basal cells, we found highly inducible *IRF1* mRNA expression by subsequent poly(I:C) stimulation, but this induction was not different between naïve and trained cells (P= n.s., **Figure 3C**).

By contrast, trained cells expressed a 4 ± 1-fold elevation of *BST2* mRNA over that of naive cells in the unstimulated conditions (P<0.001, n=3, **Figure 3D**). Interestingly, *BST2* mRNA levels were higher in trained cells in response to acute poly(I:C) stimulation than that of naïve cells (P<0.001, n=3, **Figure 3D**). Similarly, a 2 ± 0.2-fold induction of *IFITM1* mRNA was exhibited by trained cells over that of naive cells in the absence of stimulation (P<0.001, n=3, **Figure 3E**). Consistently, a 1.3 ± 0.1-fold induction of *OAS2* mRNA in trained cells was also seen over that of naive cells (P<0.001, n=3, **Figure 3F**). Consequently, the training effect on tonic expression of the intrinsic IIR program is also observed in primary human basal cells, consistent with the hSAEC model.

### 3.4 Innate training activates epithelial mesenchymal plasticity programs

**Figure 4:**
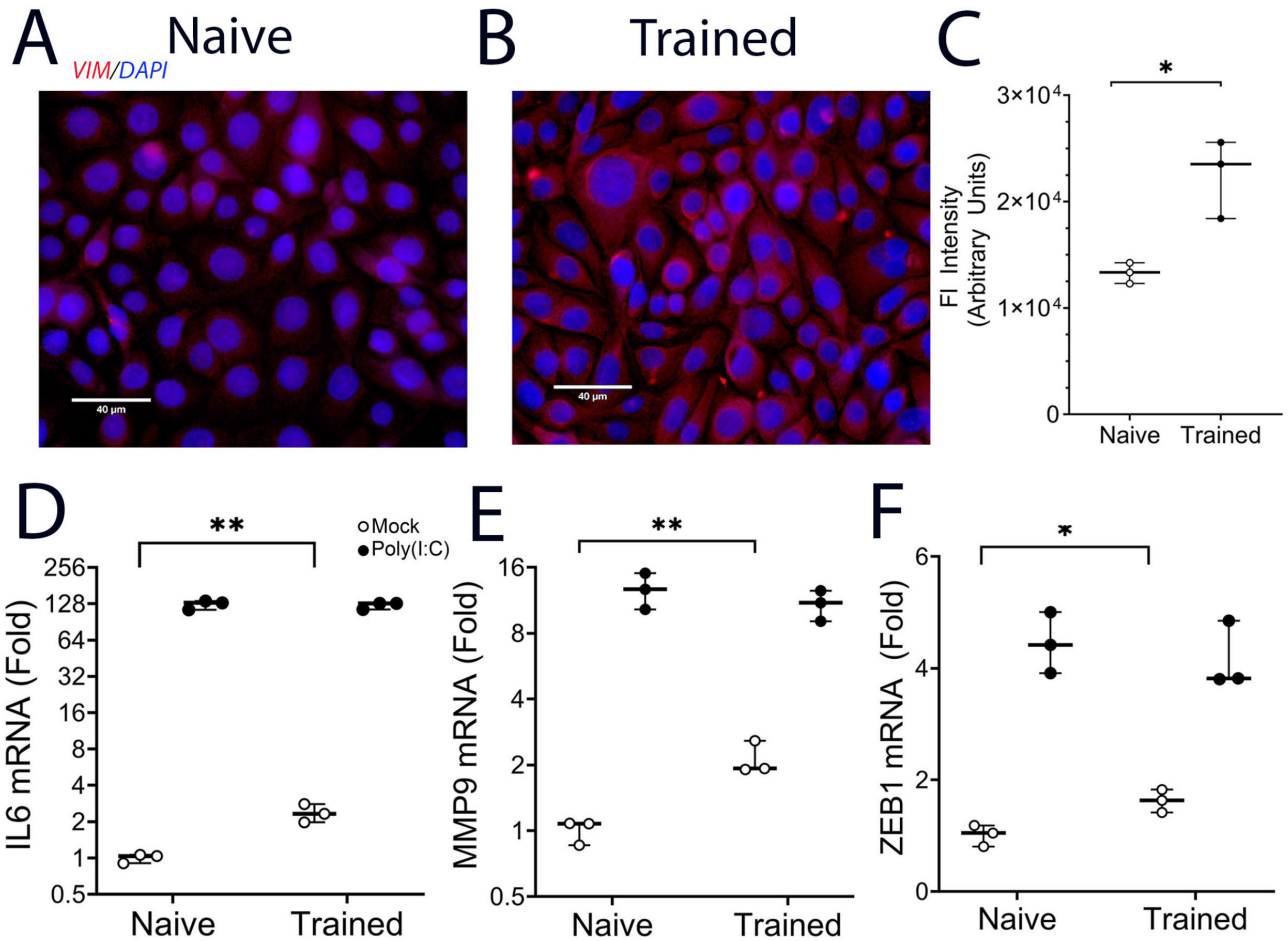
Innate training activates the EMP gene program. hSAECs were subjected to innate training as in **Figure 1**. **(A, B)** Immunofluorescence microscopy of VIM. Cells were stained with anti-VIM (red) and nuclei stained with DAPI (blue). Scale bar, 40 microns. **(C)** Fluorescence intensity of multiple independent fields for VIM was measured in Fiji. *, P<0.05. **(D–F)**, Naïve or trained cells were stimulated in the absence (open circles) or presence of poly(I:C)(filled circles) and Q-RT-PCR conducted. Shown is fold change mRNA normalized to PPIA as internal control. **(D)** IL6 mRNA; **(E)** MMP9 mRNA; **(F)** ZEB1 mRNA. Each symbol is an independent replicate. *P<0.05; **P< 0.01 *post-hoc*.

Our previous work has found that chronic TLR3 activation over a 3 week stimulation triggers airway basal cells to enter stable mesenchymal transition (EMT) via an NF-κB-dependent mechanism, resulting in growth factor secretion, extracellular matrix production and acquisition of cellular motility (37, 38). Stable EMT is preceded by a series of partial mesenchymal transitions, EMP, initiated by disrupting a double negative feedback loop with ZEB1, miRNAs and SNAI that activate mesenchymal intermediate filament vimentin (VIM) expression and repress epithelial cadherin expression (39). However the effect of short term TLR3 activation on initial entry into EMP is unknown. To determine if short-term training induces mesenchymal plasticity, IFM was performed measuring VIM expression. Here, we observed that training resulted in characteristic cell shape changes (flattening, elongation) and a 1.7-fold increase in VIM staining in the cytoplasm, consistent with early entry into EMP (n=3, P<0.05; **Figures 4A, B**, quantified in **Figure 4C**).

To better understand this phenomenon, we conducted additional Q-RT-PCR studies on the EMP transcriptional program. We observed that innate training activated expression of the growth factor interleukin 6 (IL6) mRNA by 2.4 ± 0.4-fold over that of naive hSAECs (P<0.01, n=3, **Figure 4D**). Matrix metalloproteinase 9 (MMP9) was similarly increased to 2.1 ± 0.4-fold over that of naive cells (P<0.01, n=3, **Figure 4E**), and the mesenchymal transcription factor, ZEB1, was 1.6 ± 0.2-fold over that of naive cells (P<0.01, n=3, **Figure 4F**). These data indicated that TLR3-dependent training couples initiation of EMP with the intrinsic IIR.

### 3.5 Innate memory requires the IRF1 transcription factor

Our previous IFM and western blot analyses showed that nuclear IRF1 is constitutively expressed in hSAECs, where its ambient levels are required for intrinsic IIR gene expression (24), but is further induced by viral patterns through a positive autoregulatory loop (18). To demonstrate its role in activation of intrinsic IIR and initiating EMP, we examined the effect of innate training in response to IRF1-knock down (KD), produced by CRISPR/Cas9 genome editing. Small guide RNA (sgRNA) #3 was the most effective of the guide RNAs screened and reduced constitutive and RSV inducible IRF1 mRNA in sgRNA-transfected hSAECs (24).

**Figure 5:**
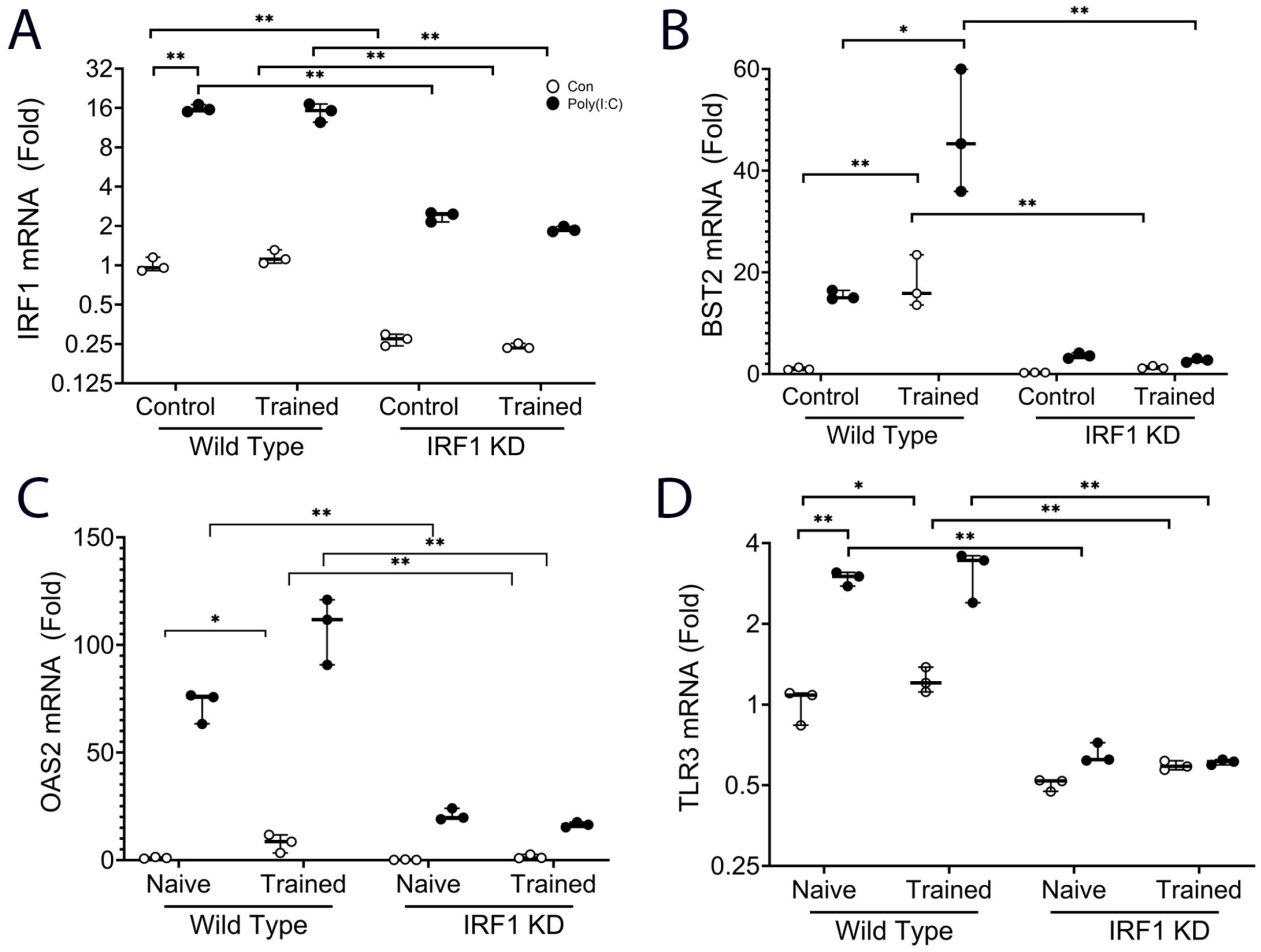
IRF1 mediates innate training. Wild type (WT) or IRF1 KD hSAECs were subjected to innate training and stimulated in the absence (open circles) or presence of poly(I:C) (filled circles). **(A–D)** Q-RT-PCR of fold change mRNA normalized to *PPIA* as internal control. **(A)** *IRF1* mRNA; **(B)** *BST2* mRNA; **(C)** *OAS2* mRNA; and **(D)**, *TLR3* mRNA. Each symbol is an independent replicate. *P<0.05; **P< 0.01 *post-hoc*.

To confirm effective IRF1 KD, we observed that *IRF1* mRNA was substantially reduced to ~25% that of *IRF1* levels in wild type (WT) cells either the naïve or trained states (P<0.01, n=3, **Figure 5A**). After poly(I:C) stimulation, *IRF1* mRNA levels in the IRF1 KDs were reduced to ~15% that of WT cells (P<0.01, n=3, **Figure 5A**), confirming effective inhibition (interestingly, alternatively spliced, nonfunctional IRF1 transcripts lacking the DNA binding domain are expressed by the IRF1 KD cells, which are not translated into functional protein (40), and data not shown).

Using this model, we were poised to examine the requirement of IRF1 for intrinsic memory. In WT naïve cells, *BST2* mRNA is robustly increased by 15 ± 1-fold in response to acute TLR3 stimulation (P<0.01, n=3, **Figure 5B**). In the WT background, training induced a memory response with a 17.6 ± 5.2-fold induction of *BST2* mRNA levels of trained vs naïve unstimulated cells (P<0.01, n=3, **Figure 5B**). We further observed the trained WT cells showed a 3-fold potentiation of *BST2* mRNA expression to 47 ± 12-fold over that seen in naïve WT cells when subjected to TLR3 stimulation (P<0.05, n=3, **Figure 5B**), indicating that TLR3 training potentiates the intrinsic IIR.

In marked contrast, the training induction of *BST2* mRNA was substantially reduced in IRF1 KD cells to 1.3 ± 0.3-fold that of naïve WT cells (P<0.01, n=3, **Figure 5B**). Additionally, the robust induction produced by acute TLR3 activation was substantially reduced in IRF1 KD cells, reaching only 3.5 ± 0.5-fold that of naïve WT cells (P<0.01, n=3, **Figure 5B**). These effects indicated that innate memory is dependent on IRF1 signaling.

The response of intrinsic IIR genes *OAS2* and *TLR3* were qualitatively similar to that of *BST2*, where TLR3 training induced robust basal expression over that of naïve cells, and were further potentiated by TLR3 activation (**Figures 5C, D**). Importantly, in the IRF1 KD cells, the training effect of increased expression of *OAS2* and *TLR3* mRNA in the unstimulated condition were completely lost. In a like fashion, the robust potentiation of expression by acute poly(I:C) stimulation was also significantly reduced (**Figures 5C, D**). These data indicate that training resulted in enhanced basal expression, a potentiated intrinsic IIR, and that IRF1 signaling is absolutely required for this effect.

### 3.6 Initiation of the EMP program is IRF1-dependent

**Figure 6:**
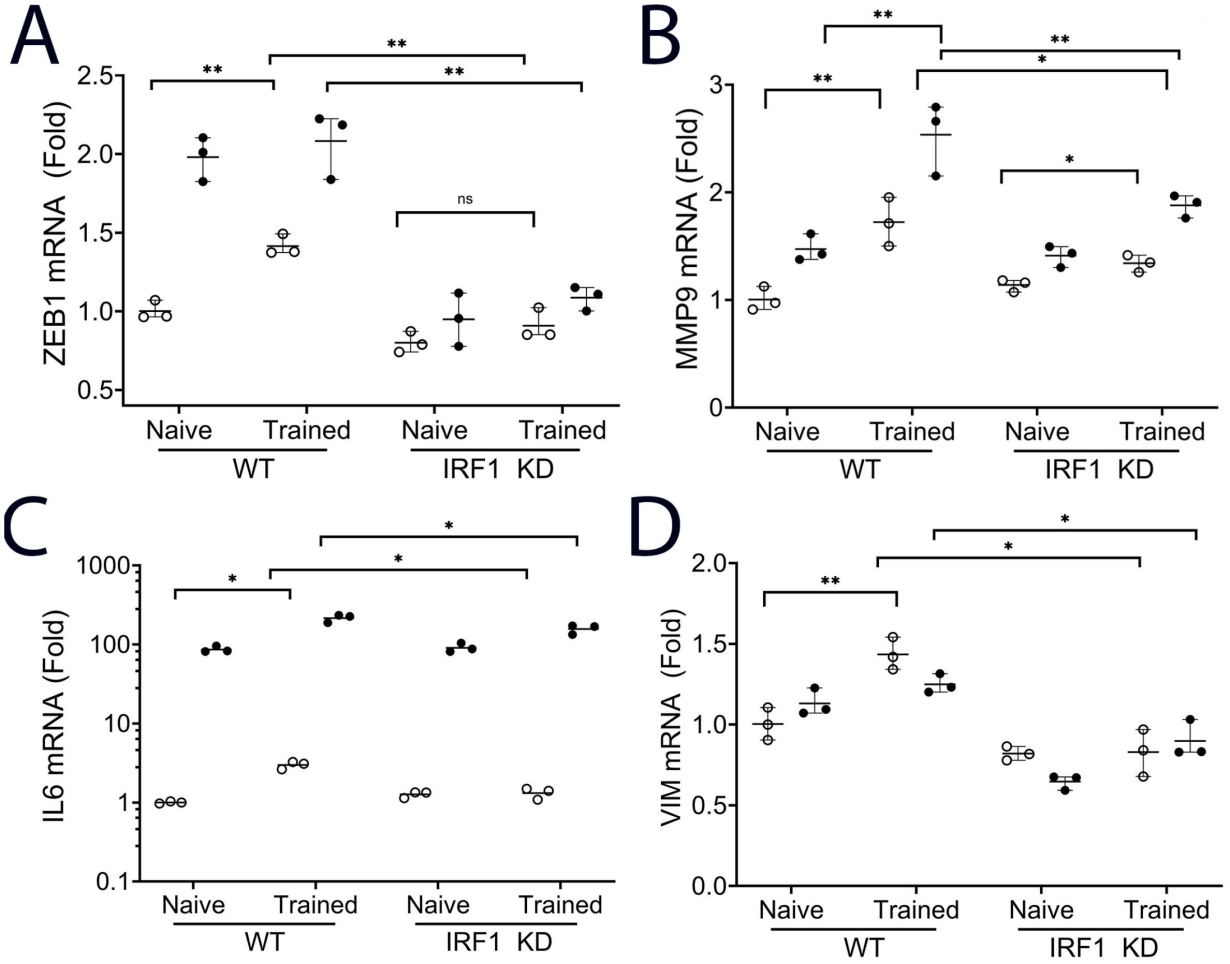
IRF1 activation mediates EMP in trained cells. Wild type (WT) or IRF1 KD hSAEC were subjected to innate training and stimulated in the absence (control, open circles) or presence of poly(I:C)(filled circles). **(A–D)** Q-RT-PCR of fold change mRNA normalized to PPIA as internal control. **(A)** ZEB1 mRNA; **(B)** MMP9 mRNA; **(C)** IL6 mRNA; **(D)** VIM mRNA. Each symbol is an independent replicate. *P<0.05; **P< 0.01 *post-hoc*.

We next examined the effect of IRF1 KO on the activation of the EMP network. Here we observed, the 1.4 ± 0.07-fold increase in *ZEB1* mRNA induced by training in the WT cells was reduced to 0.95 ± 0.17-fold in the IRF1 KD (both P<0.01, n=3, **Figure 6A**). And similarly, the basal upregulation of *MMP9, IL6* and *VIM* mRNAs produced by training in WT cells was largely abolished in the IRF1 KD cells (all P<0.05, n=3, **Figures 6B–D**).

Collectively, these data indicate that innate memory is mediated by IRF1 activation, coordinately enhancing intrinsic IIR and initiating EMP gene programs. Moreover, the use of a nonreplicating TLR3 agonist showed that memory is characterized by potentiated TLR3 response.

### 3.7 IRF1 is recruited into the BRD4 chromatin remodeling complex

**Figure 7:**
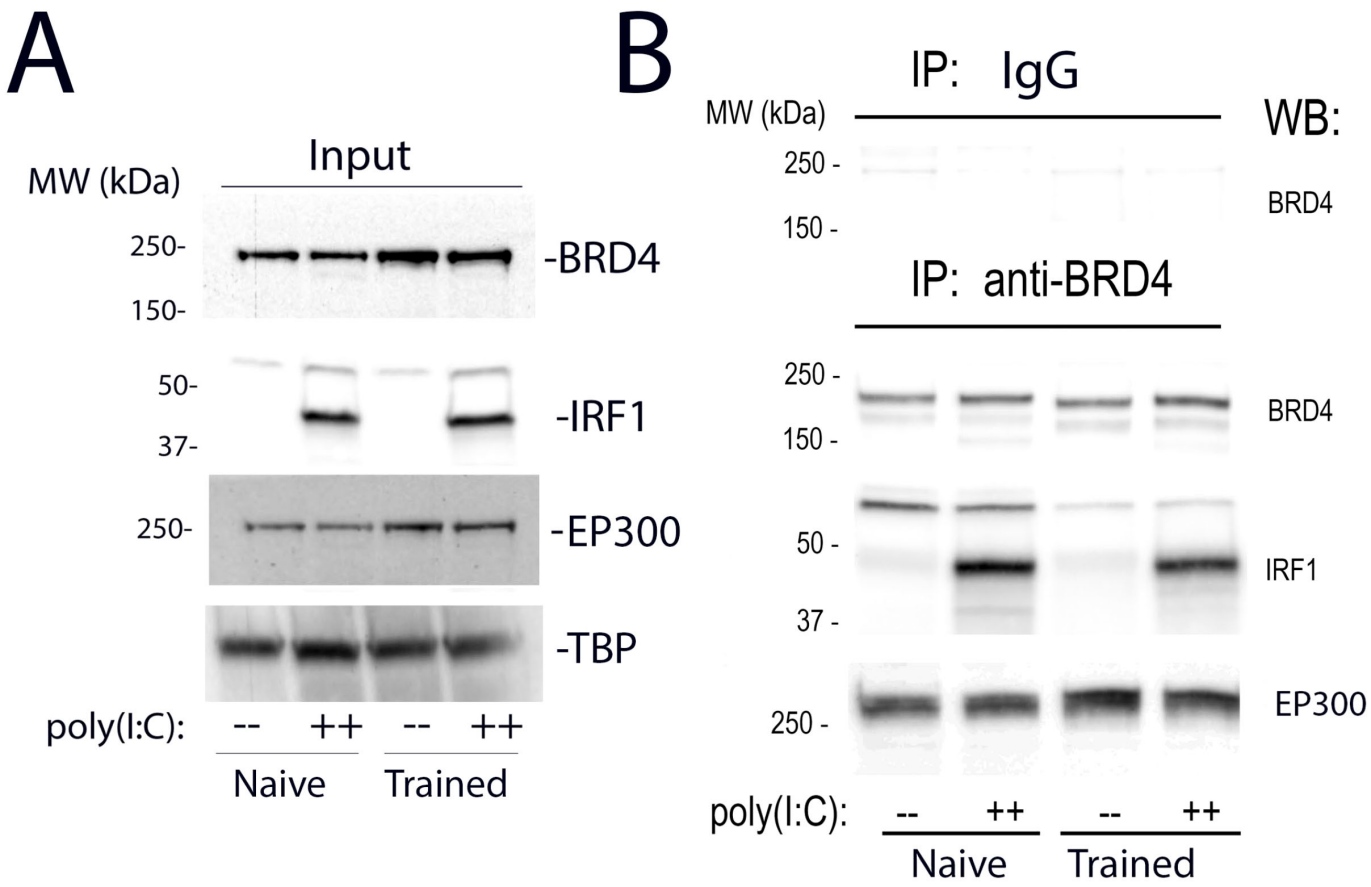
Effect of innate training on the BRD4 complex. WT hSAECs subjected to TLR3 training and acutely stimulated in the absence or presence of poly(I:C). **(A)** Nuclear extracts were prepared and analyzed by WB. Membranes were probed for abundance of BRD4, IRF1 and EP300. MW, molecular weight (kDa). TATA box binding protein (TBP) was used as housekeeping control. Note the similar level of induction of IRF1 by poly(I:C) treatment in both naive and trained conditions. **(B)** Extracts were subjected to native immunoprecipitation with IgG as control or anti-BRD4 Ab and fractionated on SDS-PAGE. Western immunoblot (WB) were performed for the indicated targets shown at right. Note the poly(I:C) highly increases IRF1 incorporation into the BRD4 CRC to a similar extent in naïve and trained cells. By contrast, EP300 binding is slightly increased in the trained vs naïve condition and not detectably changed by poly(I:C) stimulation.

Our previous work has shown that the BRD4 chromatin remodeling complex (CRC) couples NFκB activation to a sustained IRF1 amplification loop in response in acute innate signaling (41). Here, the BRD4 CRC facilitates regulated transcriptional elongation (26, 42). by forming an extensive protein interaction network that includes the histone acetyltransferase, EP300/p300. Upon recruitment to IIR genes, the BRD4 CRC regulates chromatin conformation enhancing transcription factor accessibility as well as RNA processivity, transitioning RNA Pol II to an activated form, rapidly transcribing target genes (24). We asked whether training affected the abundance of IRF1 or its interaction with BRD4 CRC. Nuclei from naïve or trained WT hSAECs in the absence or presence of acute TLR3 activation were prepared and analyzed by Western Blot (WB). We observed the steady state abundance of ~200 kDa isoform of BRD4 was slightly ~2-fold increased by training (**Figure 7A**). In striking contrast, the abundance of IRF1 was ~10-fold inducible by acute TLR3 stimulation in both the naïve and trained nuclei (**Figure 7A**). Training slightly increased the abundance of the >250 kDa isoform of EP300 (**Figure 7A**).

To determine whether innate training affected BRD4 CRC, we conducted nondenaturing coimmunoprecipitation (CoIP) experiments. Naïve or trained nuclei were IPed with either IgG or anti-BRD4 Ab, and the composition of the BRD4 CRC quantitated by WB. Relative to IgG, BRD4 Ab produced a significant enrichment of BRD4 signal, which was not different between naïve vs trained cells either in the absence or presence of TLR3 activation (**Figure 7B**). Strikingly, acute TLR3 activation induced a robust induction of IRF1 within the BRD4 CRC in both naive and trained cells (**Figure 7B**). Finally, we observed training produced a ~2-fold enrichment of EP300 in the BRD4 CRC, seen both in the absence and presence of poly(I:C) stimulation. Collectively we conclude that IRF1 is highly incorporated into the BRD4 CRC as a consequence of training.

### 3.8 Innate training does not affect constitutive or inducible IRF1-BRD4 binding

We next explored whether training affected IRF1 binding to target innate IIR promoters within their native chromatin environment using chromatin immunoprecipitation (XChIP). Control or trained cells were stimulated in the absence or presence of poly(I:C) and promoter enrichment of IRF1 and BRD4 accumulation first determined by XChIP.

Examining first IRF1 binding, we previously demonstrated that IRF1 antibody produces a significant ~10-fold increase in target chromatin over that produced by IgG (24), so IRF1 signals in these assays provide quantitative information on both basal and inducible IRF1 binding.

**Figure 8:**
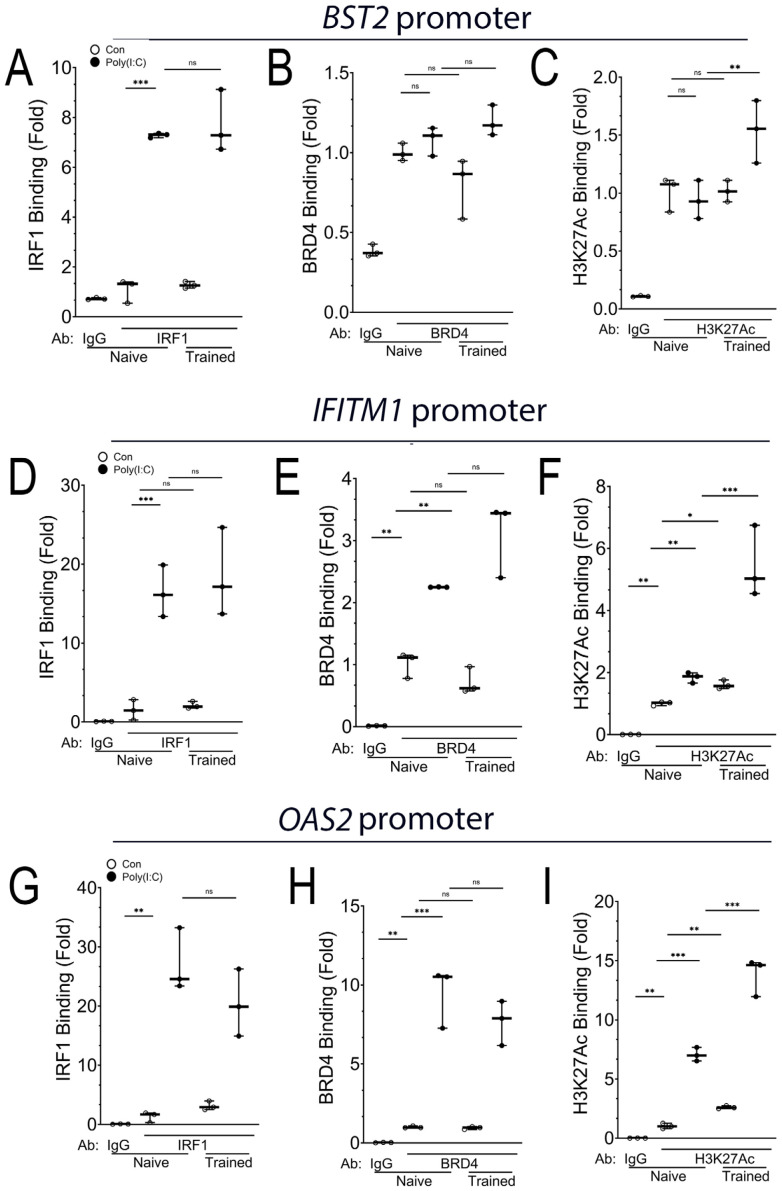
Innate training produces enhanced H3K27ac responses. Wild type (WT) hSAECs cells were subjected to innate training and stimulated in the absence (control) or presence of poly(I:C). Shown are Q-gPCR of chromatin Immunoprecipitation (XChIP) normalized to input DNA and expressed as fold change over control. **(A–C)** Q-gPCR for BST2 promoter. **(D–F)** Q-gPCR for IFITM1 promoter. **(G–I)** Q-gPCR for OAS2 promoter. **(A, D, G)** IP with IRF1 Ab; **(B, E, H)** IP with BRD4 Ab; and **(C, F, I)** IP with H3K27ac Ab. Each symbol is an independent replicate; n.s., not significant; *P<0.05; **P< 0.01; ***P< 0.001, *post-hoc*.

Interestingly, although we observed acute TLR3 stimulation produces a 7.2 ± 0.1-fold increase in IRF1 binding to the BST2 promoter relative to mock treatment in naive cells (P<0.05, n=3, **Figure 8A**), basal IRF1 binding was not induced in the unstimulated trained cells (**Figure 8A**). And, also, importantly, the abundance of IRF1 was not detectably increased by acute TLR3 stimulation in the trained cells (**Figure 8A**), despite the robust activation of the mRNA.

Next, examining BRD4 binding to the *BST2* promoter, a ~4-fold increase in BRD4 abundance was observed in unstimulated cells relative to the IgG signal, indicating that BRD4 constitutively binds this gene in naïve cells (**Figure 8B**). And, although *BST2* mRNA is highly induced by TLR3 stimulation in a BRD4-dependent manner, we found that acute stimulation did not change the amount of BRD4 abundance in naïve cells (**Figure 8B**). Moreover, despite the requirement for BRD4 in *BST2* mRNA expression during training, we found training did not change the abundance of BRD4 binding, nor was there increased BRD4 binding in the trained cells after acute TLR3 stimulation (**Figure 8B**).

Examining IRF1 binding to the *IFITM1* promoter, we observed that, although poly(I:C) induced a robust 16.4 ± 3.2-fold increase in IRF1 binding relative to mock treatment in naïve cells (P<0.01, n=3, **Figure 8D**), there was no difference in IRF1 binding between the unstimulated trained cells and the unstimulated naïve cells, nor in the 18-fold induction of IRF1 in TLR3 stimulated trained cells relative to the 16-fold induction in TLR3-stimulated naïve cells (n.s., n=3, **Figure 8D**).

Analyzing BRD4 binding to the *IFITM1* promoter, we also noted BRD4 binding to the *IFITM1* promoter was not different between the naïve and trained unstimulated cells (**Figure 8E**). And although TLR3 stimulation induced ~2.4-fold increase in BRD4, this pattern of induction was not detectably different in the trained cells (**Figure 8E**).

Analysis of IRF1 binding to the *OAS2* promoter showed a similar pattern, where acute poly(I:C) stimulation induced a 27.1 ± 5.4-fold increase in IRF1 binding over that of unstimulated naive cells (P<0.01, n=3, **Figure 8G**). We noted that IRF1 binding was not different in the unstimulated trained cells vs unstimulated naïve cells, nor in the poly(I:C) stimulated trained cells vs the poly(I:C) stimulated naïve cells (n.s., n=3, **Figure 8F**). And for BRD4 binding, we observed acute TLR3 stimulation induced a 9.5 ± 1.9-fold increase in naive cells (P<0.01, n=3, **Figure 8H**) but the amount of BRD4 binding to unstimulated naïve cells was not different from unstimulated naïve cells. Similarly, poly(I:C) stimulated BRD4 binding was not different in the trained vs naïve cells (n.s., n=3, **Figure 8H**). Collectively, although IRF1 and BRD4 are induced by acute TLR3 activation to bind to the intrinsic IIR genes, the increased basal gene activation in the trained state could not be explained by enhanced IRF1-BRD4 binding.

### 3.9 Innate training enhances basal and inducible H3K27ac at regulated regions

Informed by previous work in leukocytes that cell memory involved the formation of open chromatin domains, we explored this mechanism in basal epithelial cell model. We therefore analyzed acetylated histone H3 (H3K27ac), an epigenetic histone mark associated with activated chromatin in open chromatin regions (43), in the same XChIP experiment. Examining first *BST2*, H3K27ac binding was substantially abundant in unstimulated naïve cells, where a >10-fold increase in H3K27ac signal was produced relative to IgG control (**Figure 8C**). Interestingly acute poly(I:C) stimulation did not change H3K27ac binding in naïve cells, nor was there a change in H3K27ac after training (**Figure 8C**). However, poly(I:C) induction increased a 1.54 ± 0.3-fold increase in H3K27ac binding after training (P<0.05, n=3, **Figure 8C**).

Examining H3K27ac binding to *IFITM1*, we noted acute TLR3 activation induced a 1.8 ± 0.2-fold increase in H3K27ac levels in naïve cells that was slightly enhanced by training, while a robust 5.4 ± 1.2 increase was seen in the poly(I:C)-stimulated trained cells (P<0.001, n=3, **Figure 8F**). And although acute TLR3 stimulation increased H3K27ac binding to *OAS2* promoter by 7.1 ± 0.6-fold, a potentiated 13.8 ± 1.6-fold binding was observed in the trained cells (P<0.01, n=3, **Figure 8I**). These data indicate that training primed the intrinsic IIR gene environment to more robustly transition into activated, open chromatin upon TLR3 activation.

### 3.10 Innate training requires BRD4 acetyltransferase activity

We next explored whether BRD4 played a functional role in IRF1-dependent innate training, focusing on the central role of BRD4’s bromodomains (BDs) a primary point of inducible interaction with acetylated transcription factors and histones (44). We examined the effect of a BRD4 selective bromodomain inhibitor that competitively disrupts both BD1 and BD2 using a well-characterized BRD4 inhibitor (BRD4i), ZL0969 (29). WT hSAECs were untreated (naïve) or trained in the presence of the vehicle (solvent) or BRD4i (10 µM), resulting in 4 treatment groups. Each group was then divided into two, one unstimulated and the second TLR3 stimulated followed by measurement of the IIR by Q-RT-PCR.

**Figure 9:**
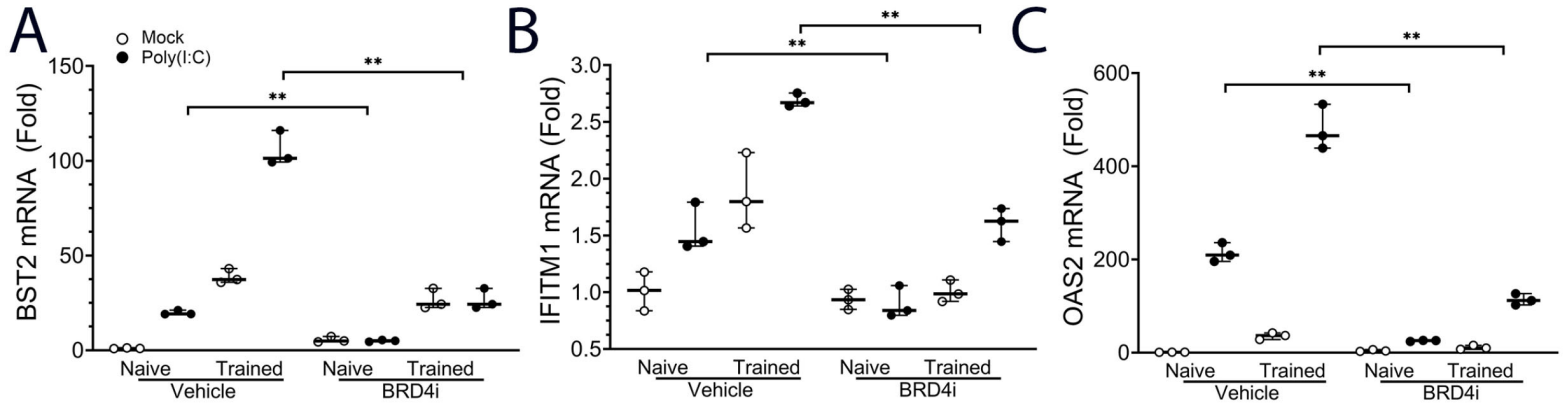
BRD4 histone acetyltransferase activity is required for acquiring memory. Wild type (WT) hSAECs cells were subjected to innate training in the presence of vehicle or ZL0969 (BRD4i) and stimulated in the absence (open circle) or presence of poly(I:C) (closed circle). **(A–C)** Q-RT-PCR of fold change mRNA normalized to *PPIA* as internal control. **(A)** *BST2* mRNA; **(B)** *IFITM1* mRNA; **(C)** *OAS2* mRNA. Each symbol is an independent replicate; **P< 0.01 *post-hoc*.

We observed the characteristic pattern of *BST2* mRNA expression was produced in vehicle-treated cells in response to TLR3 training. In unstimulated cells, *BST2* mRNA was increased 39 ± 3.9-fold by training (P<0.01, n=3, **Figure 9A**). And although BST2 mRNA was induced to 19.8 ± 1.2-fold by acute TLR3 stimulation in vehicle-treated naïve cells, this induction was potentiated by the training where a 106 ± 9-fold activation was seen (P<0.01, n=3, **Figure 9A**).

By contrast, in the BRD4i treated naïve cells, we observed *BST2* mRNA induction by acute TLR3 activation was completely inhibited by BRD4i treatment (P<0.01, n=3, **Figure 9A**), consistent with our previous observations using a related BRD4i (45). In the BRD4i-treated trained cells, *BST2* mRNA induction by acute TLR3 stimulation was also completely inhibited, where no difference could be observed between unstimulated and TLR3-stimulated cells (n.s., n=3, **Figure 9A**). BRD4i similarly substantially reduced the effect of training on constitutive *IFITM1* expression as well as that produced by acute poly(I:C) stimulation (both P<0.01, n=3, **Figure 9B**). A similar effect was produced in *OAS2* mRNA expression in naïve as well as trained cells (**Figure 9C**) (P<0.01 comparisons of TLR3 stimulated by treatment group, n=3). Collectively these findings indicated BRD4 BD interactions were required for acquisition of memory and potentiation of TLR3 signaling in the intrinsic IIR.

### 3.11 BRD4 BD is required for the training induction of H3K27ac formation

Building on our findings that the BRD4 BD is functionally required for training, we examined the effect of the BRD4i on BRD4 and H3K27ac recruitment. WT hSAECs were trained in the absence or presence of BRD4i and each group left unstimulated or stimulated with poly(I:C). XChIP was then performed for BRD4 and H3K27ac.

**Figure 10:**
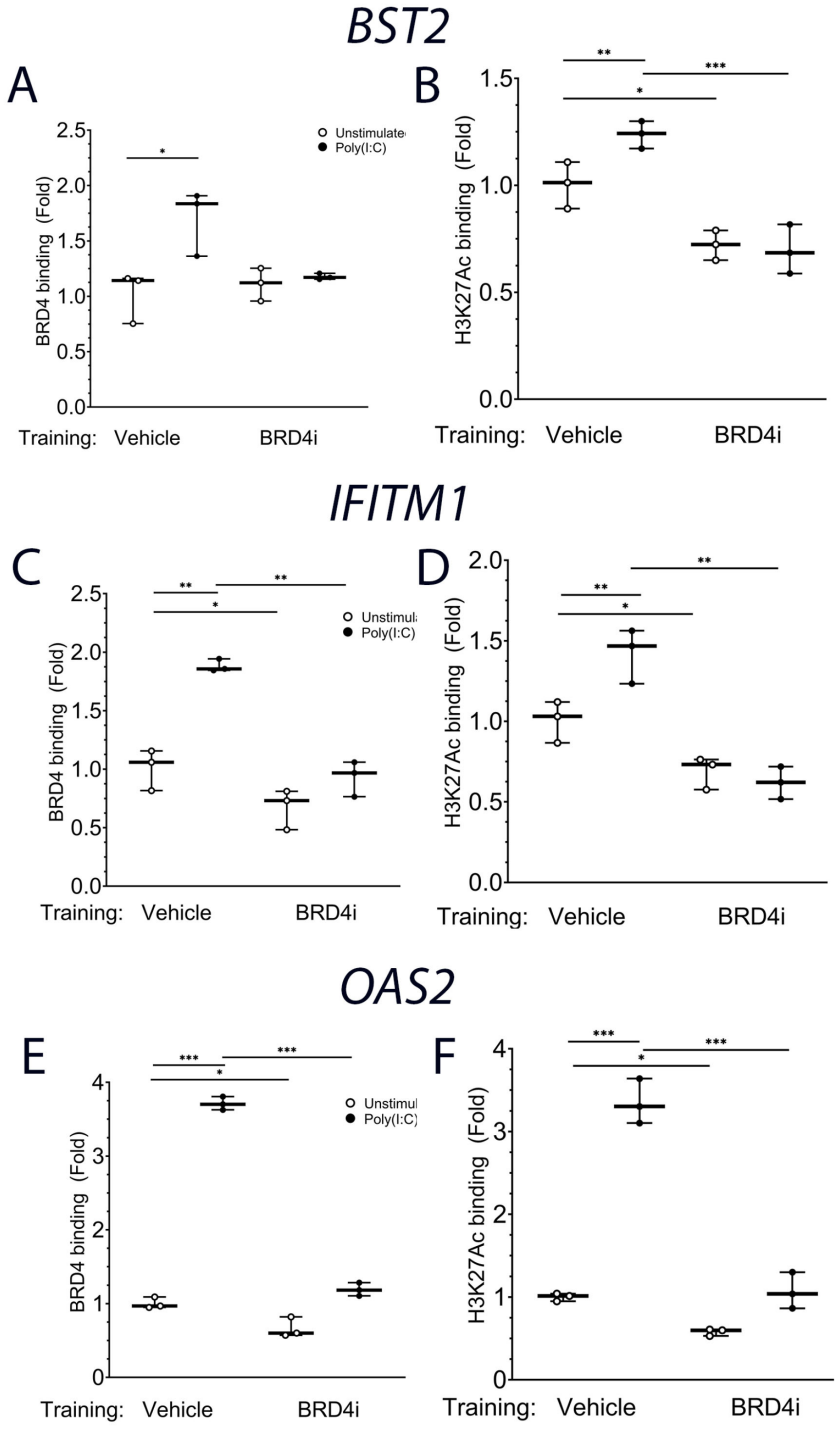
BRD4 mediates innate training and H3K27Ac induction. Wild type (WT) hSAECs cells were subjected to innate training in the presence of vehicle or BRD4i. Cells were then stimulated in the absence (open circle) or presence of poly(I:C) (closed circle). Shown are Q-gPCR of XChIP normalized to input DNA and expressed as fold change over control. **(A, B)** *BST2* promoter; **(C, D)** *IFITM1* promoter; **(E, F)** *OAS2* promoter. **(A, C, E)** IP with BRD4 Ab; **(B, D, F)** IP with H3K27ac Ab. Each symbol is an independent replicate; *P<0.05; **P< 0.01; ***P< 0.001, *post-hoc*. Note the reduced H3K27 Ac binding in the BRD4i treated cells.

On *BST2*, we observed that acute poly(I:C) increased BRD4 binding 1.7 ± 0.2-fold (P<0.05, n=3, **Figure 10A**) that was completely inhibited by the BRD4i treatment (P<0.01, n=3, **Figure 10A**). And strikingly, BRD4i treatment blocked both the basal and poly(I:C) induced H3K27Ac formation on BST2 (P<0.001, n=3, **Figure 10B**).

A similar dependence for BRD4 was observed on the *IFITM1* promoter, where the 1.9 ± 0.05-fold increase of BRD4 binding by acute poly(I:C) induction was reduced to that of unstimulated cells (P<0.01, n=3, **Figure 10C**). As before, the induction of H3K27ac was similarly blocked by BRD4i treatment (P<0.01, n=3, **Figure 10D**).

Finally, the 3.7 ± 0.1- fold increase in BRD4 binding to *OAS2* by acute poly(I:C) stimulation was substantially inhibited by BRD4i (P<0.01, n=3, **Figure 10E**). Similarly, the 3.3 ± 0.3-fold increase in H3K27Ac by acute poly(:C) stimulation was completely inhibited by BRD4i treatment (P<0.01, n=3, **Figure 10F**). We interpreted these data to indicate that BRD4 recruitment to intrinsic IIR genes required BRD4 BD interactions, and that BRD4 was required for the induction of H3K27ac formation.

### 3.12 Innate training enhances EP300 loading on intrinsic IIR genes

Although BRD4 is functionally required for the effects of training, we noted the presence of the EP300 histone acetyltransferase within the BRD4 CRC in our Co-IP. EP300 is a potent H3K27 acetyl transferase that may explain the effect of BRD4 on H3K27Ac formation (46). For this, naïve and trained cells were analyzed for changes in EP300 by XChIP.

**Figure 11:**
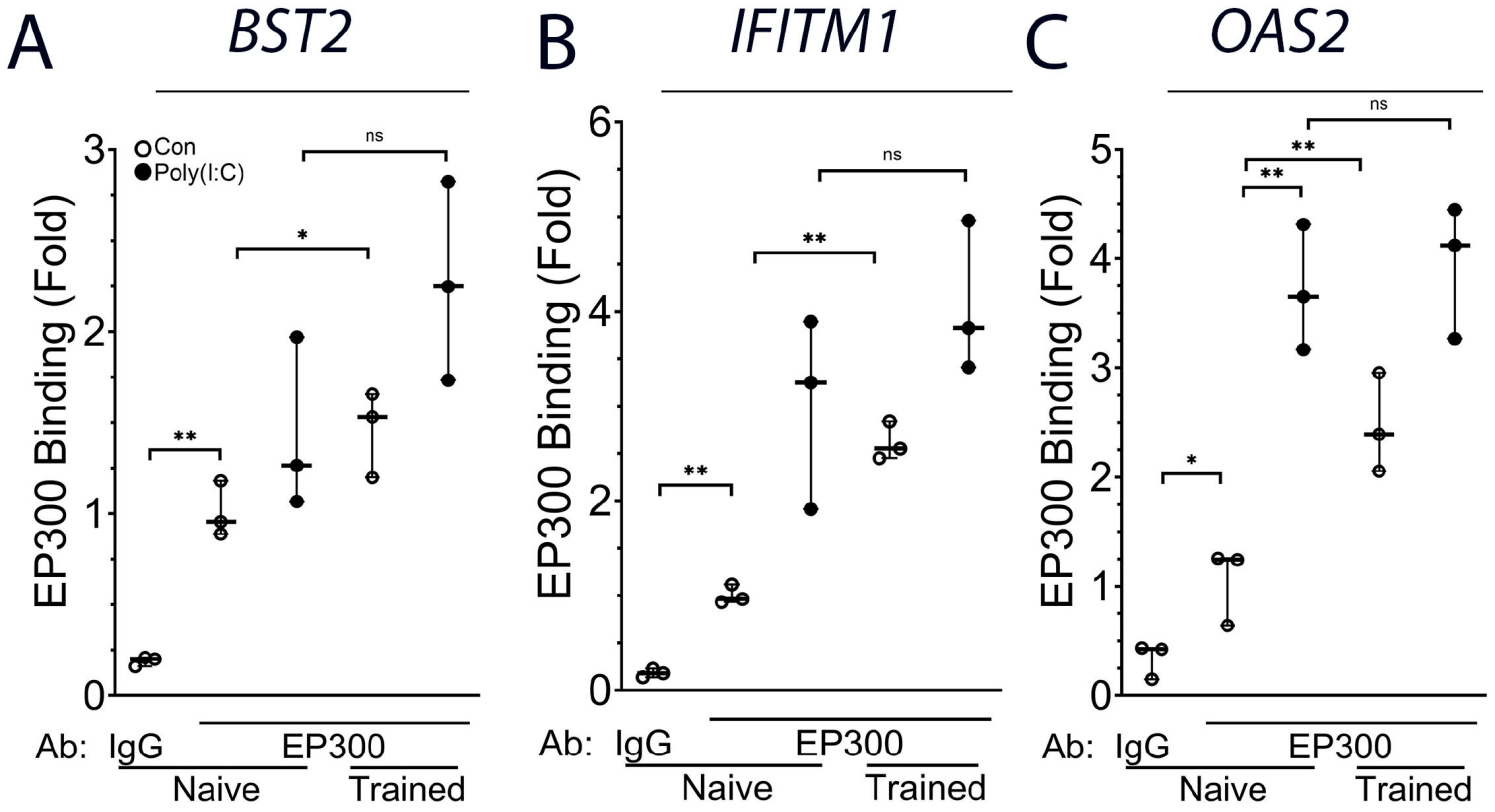
Innate training enhances EP300 binding. Wild type (WT) hSAECs cells were subjected to innate training and stimulated in the absence (control) or presence of poly(I:C). XChIP experiments are performed for EP300 binding normalized to input IgG and expressed as fold change over control. **(A)** *BST2*; **(B)** *IFITM1*; **(C)** *OAS2* promoter. Each symbol is an independent replicate; n.s., not significant; *P<0.05; **P< 0.01 *post-hoc*. Note the increased EP300 in the unstimulated and stimulated cells as a result of training.

We found that relative to IgG, IP with anti-EP300 Ab produced a 5-fold increase in *BST2* promoter XChIP signal, indicating EP300 constitutively binds to *BST2* (P<0.01, n=3, **Figure 11A**). In naïve cells, acute TLR3 signaling did not produce a detectable induction of EP300 binding (**Figure 11A**). By contrast, in unstimulated trained cells, a significant 1.4 ± 0.2-fold increase in EP300 binding was seen relative to naïve cells (P<0.05, n=3, **Figure 11A**). In acutely TLR3-stimulated trained cells, a more significant 2.3 ± 0.5-fold increase in EP300 binding was seen; this was not different from TLR3-stimulated naïve cells (n.s., n=3, **Figure 11A**).

Analysis of the *IFITM1* promoter showed constitutive EP300 binding (EP300 Ab vs IgG, **Figure 9A**). In naïve cells, poly(I:C) stimulation further increased EP300 3.1 ± 1-fold (P<0.01, n=3, **Figure 11B**). We found that EP300 binding in the unstimulated trained cells was significantly increased 2.6 ± 0.2-fold over that of unstimulated naïve cells (P<0.01, n=3, **Figure 11B**). EP300 binding increased to 4 ± 0.8-fold in the TLR3-stimulated trained cells, to a level indistinguishable of that of naïve cells (P<0.001, n=3, **Figure 11B**).

Consistently, the same pattern of EP300 binding to the *OAS2* promoter was seen. Training induced a 1.5 ± 0.5-fold increase of EP300 binding to *OAS2* over that of untreated naïve cells (P<0.001, n=3, **Figure 11C**). And TLR3 stimulation in the trained cells potentiated EP300 to 4 ± 0.6-fold induction over unstimulated naïve cells, to levels that were not different from stimulated naïve cells. We therefore conclude that innate training pre-loads EP300 onto the intrinsic IIR promoters.

### 3.13 BRD4-EP300 interactions are required for H3K27 acetylation

**Figure 12:**
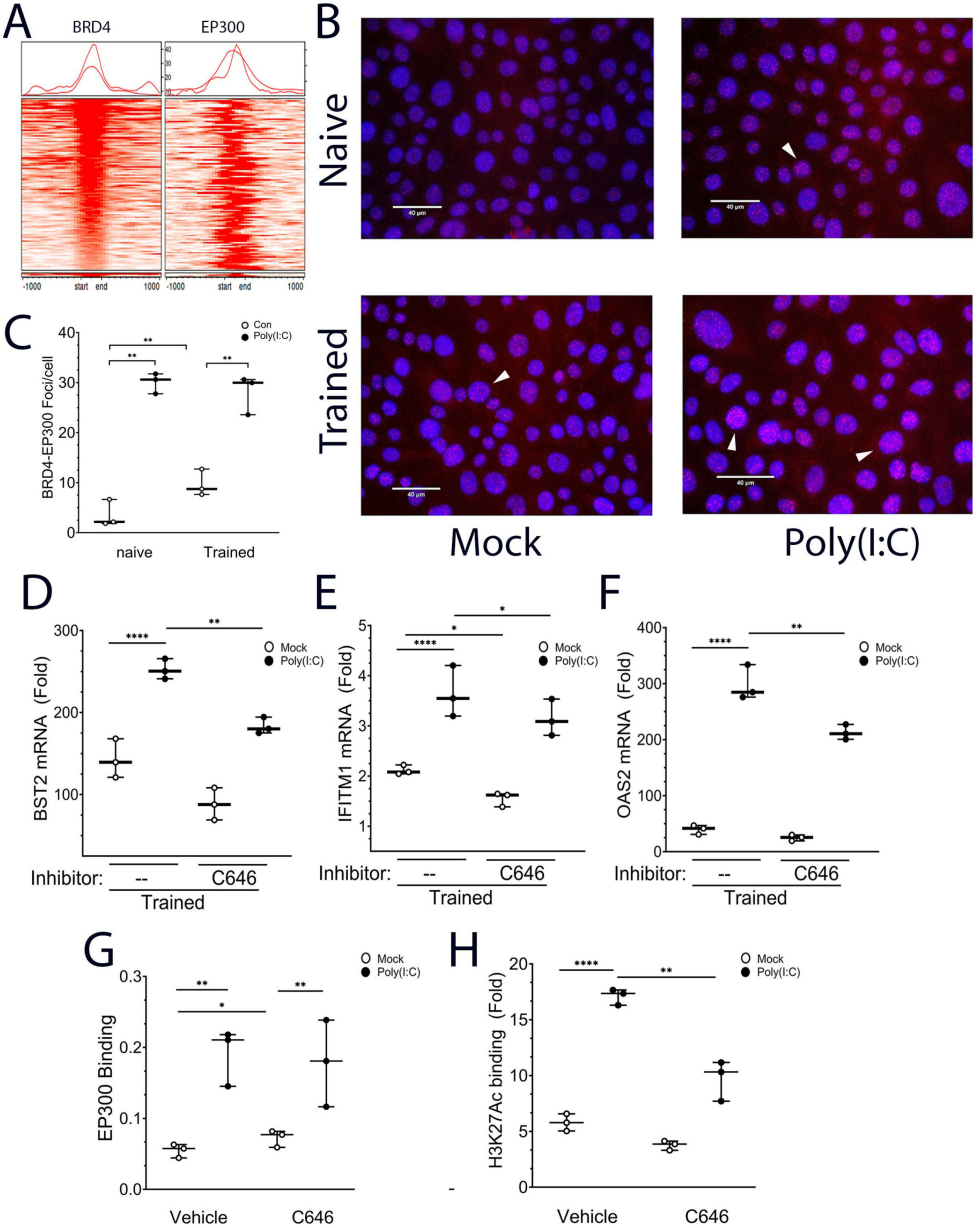
BRD4-EP300 interaction is dynamically activated by training. **(A)** Heat map of BRD4 genomic peaks and EP300 peaks from ENCODE ChIP-Seq. Peak BRD4 peaks were extracted and aligned ± 1 kb. The EP300 binding for each segment was then mapped onto the same segment. Each row represents an independent regulatory element. **(B)** Proximity ligation assays (PLAs) were performed with rabbit anti-BRD4 and mouse anti-EP300 Abs on naïve or trained cells in the absence or presence of acute poly(I:C) stimulation. After secondary antibody reaction and PCR amplification, regions of interaction are indicated by dots. Nuclei are counterstained with DAPI. Scale marker is 40 μM. Strongly staining nuclei containing BRD4 and EP300 complexes are indicated by the arrowheads. **(C)** Quantitation of nuclear BRD4-EP300 complexes by treatment type. PLAs from panel **(B)** were quantitated from >200 nuclei in >5 independent fields. Each symbol represents a complex from >200 nuclei. ****, P<.0001. **(D–F)** Effect of EP300 inhibitor on intrinsic IIR genes. Q-gPCR showing fold changes in mRNA. **(D)** *BST2*, **(E)** *IFITM1* and **(F)** *OAS2* mRNA expression. Each symbol is an independent replicate; *P<0.05; **P< 0.01 *post-hoc*. **(G)** Wild type (WT) hSAECs cells were subjected to innate training in the presence of vehicle. Cells were then Poly(I:C) stimulated in the absence (vehicle) or presence of C646. **(G)** XChIP for EP300 binding on *IFITM1* **(H)** XChIP for H3K27ac binding of *IFITM1*. Each symbol is an independent replicate; *P<0.01; **P< 0.01; ***P<0.001 *post-hoc*. Note C646 treatment inhibits poly(I:C)-induced H3K27ac binding.

To further explore our findings that training enhances BRD4-EP300 association and EP300 loading on intrinsic IIR, we first examined where BRD4 and EP300 exhibit similar binding profiles. Here, publicly available ChIP Seq data in type II-like alveolar epithelial cells was extracted and analyzed. Genes demonstrating high intensity BRD4 signals were extracted, centered and ranked in order of signal intensity. The BRD4 peaks were then aligned with EP300 signals corresponding to the same genes. These data showed that high intensity BRD4 peaks were also greatly enriched in EP300 binding, supporting a functional interaction (**Figure 12A**).

To explore this relationship further, we developed proximity ligation assays (PLAs) to visualize molecular BRD4-EP300 complexes *in situ*. Naïve or trained cells in the absence or presence of acute TLR3 stimulation were fixed and stained with rabbit anti-BRD4 and mouse anti-EP300 Abs. Each Ab is conjugated to complementary oligonucleotides that are amplified by PCR, appearing as red foci in IFM foci, indicating <40 nm atomic-distance interactions (32, 47). We observed that BRD4 and EP300 exhibited little evidence of direct protein-protein interaction in the nuclei of unstimulated naïve cells (**Figure 12B**, with quantitation in **Figure 12C**). This interaction was increased by acute TLR3 activation. By contrast, substantial foci of BRD4-EP300 interaction could be identified in trained cells (white arrowheads, **Figure 12B**). This staining was further increased upon TLR3 activation in trained cells. These data support that training promotes BRD4 and EP300 interaction.

To further understand the functional role of EP300, we examined the effect of the reversible, selective EP300 acetyltransferase inhibitor (EP300i), C646 (48) on TLR3-activated intrinsic IIR gene expression in trained cells. In a manner consistent with EP300 binding to the BST2 promoter, we observed that EP300i inhibits basal and TLR3-induced BST2 mRNA expression (P<0.01, n=3, **Figure 12D**). A similar effect of C646 was observed on IFITM1 and OAS2 mRNAs (**Figures 12E, F**).

Finally, to examine the effect of the EP300 on CRC, we conducted XChIP analysis on trained cells in the absence or presence of EP300i. In vehicle treated cells, TLR3 stimulation induced ~3-fold EP300 recruitment to the IFITM1 promoter, which was not affected by EP300i treatment (**Figure 12G**). By contrast, EP300i treatment significantly reduced H3K27 Ac recruitment (P<0.01, n=3, **Figure 12H**). Collectively we interpreted these data to indicate that enhanced H3K27ac is dependent on EP300 acetyltransferase activity downstream of BRD4.

## 4 Discussion

**Figure 13:**
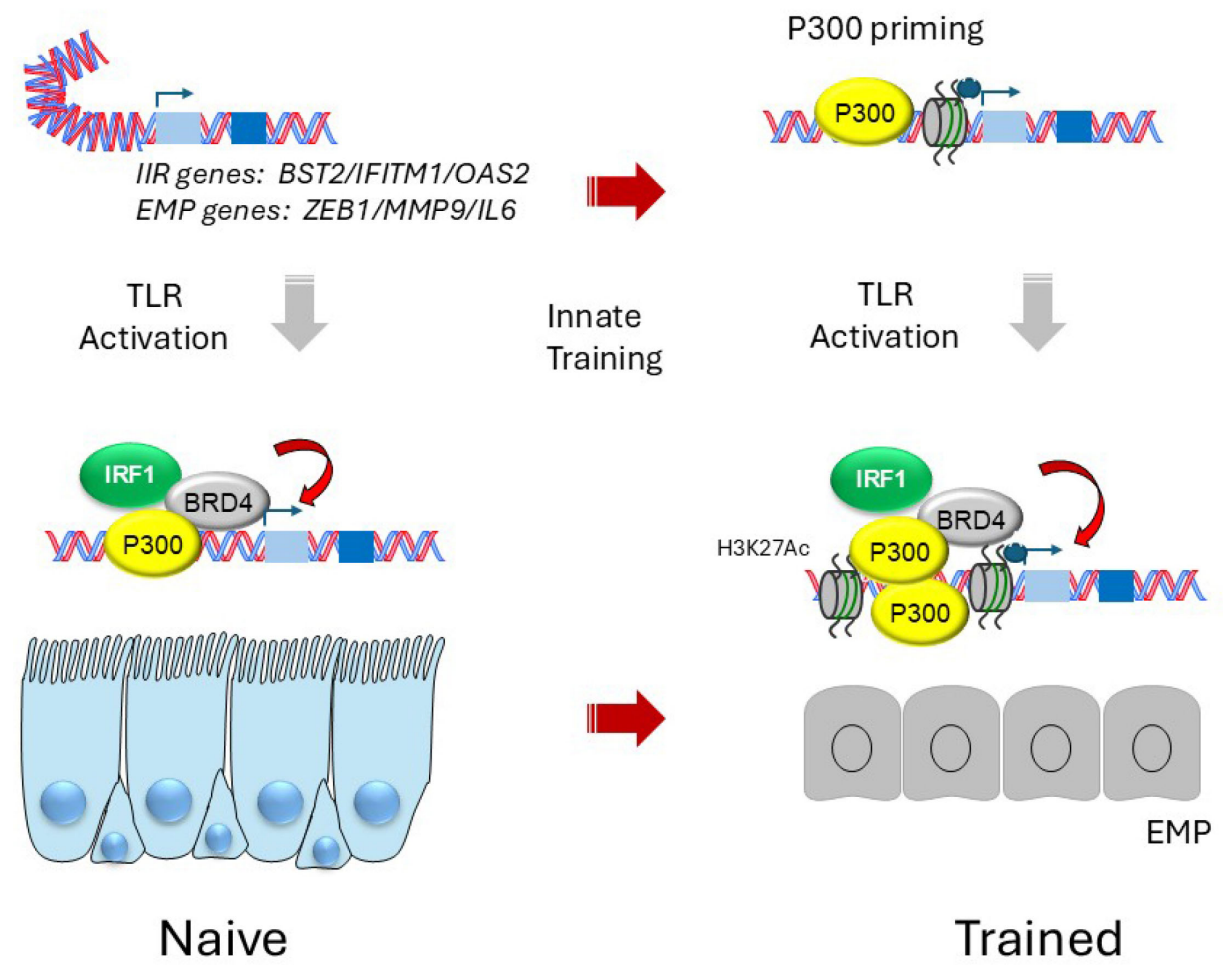
IRF1 coordinates innate training by BRD4-EP300. Schematic overview of the molecular events in innate training. In the resting condition, intrinsic IIR and epithelial mesenchymal plasticity (EMP) genes are in a condensed state with low levels of expression. In response to TLR3 activation, IRF1 activates transcription through the BRD4-EP300 complex. With repetitive stimulation, EP300 accumulates on the proximal promoters resulting in basal levels of expression. Upon stimulation, enhanced H3K27ac is induced along with increased gene expression in the trained state.

The airway epithelium is repetitively exposed to respiratory viral infections, activating innate IIR that trigger acute exacerbations and remodeling responses associated with obstructive lung disease. At the molecular level, pathogen-associated molecular patterns released during productive infection activate innate signaling leading to phenotypic mucosal adaptations. As a progenitor population of the conducting airway, the TP63^+^ airway basal cell play a central role in these adaptations. Previous work has shown that this population of cells is relatively protected from viral replication through expression of an IRF1-dependent subset of intrinsic IIR genes (24). In this study, we discover that basal cells acquire memory through modulation of the intrinsic IIR through repetitive TLR stimulation. Innate immune memory is operationally defined here as activation-enhanced expression of the IIR upon re-stimulation -a primed, poised state - rather than persistent IRF1 activation. This distinction is critical: memory implies a return to baseline followed by an amplified secondary response, whereas persistent activation reflects sustained IRF1 engagement without resolution. Our findings provide evidence for a coordinated BRD4-dependent EP300 recruitment of target promoters, potentiating their induction upon subsequent TLR3 activation. Our discovery of the enhanced H3K27ac formation is functionally mediated by BRD4 and EP300 provides insights into hyper-responsiveness. Illustrated in **Figure 13**, this study provides the first evidence for TLR3 activation of immune memory in basal airway cells with important implications for chronic disease associated with repetitive viral infections.

### 4.1 Inflammatory memory in airway epithelial cells

Immune memory has long been recognized in monocytes and macrophages, but evidence is now emerging that airway epithelial cells also acquire durable inflammatory memory. We note that Bigot and colleagues studied terminally differentiated human bronchial epithelial cells exposed to Pseudomonas aeruginosa flagellin. These cells exhibited exaggerated cytokine production upon re-stimulation (49). Joint ATAC seq and transcriptomic profiling revealed that this flagellin induced memory involved roughly 600 genes enriched for apoptosis, bacterial response, and neutrophil chemotaxis pathways (49). Although transcription factors and chromatin remodeling complexes were not experimentally validated in this study, computational analysis identified over-represented DNA binding motifs for NFIB, PRDM1, and notably IRF1. In a separate study, repeated rhinovirus (RV) infections of BEAS-2B bronchial epithelial cells produced a set of 77 “trainable” genes whose altered recall responses were linked to DNA methylation changes (50). Rhinovirus unlike RSV, is an activator of TLR2 pathways, producing a distinct spectrum of transcription factor activation (51). Together, these findings indicate that repetitive stimulation reprograms subsequent inflammatory responses of airway epithelial cells. However, the downstream gene regulatory networks are stimulus-dependent and the molecular mechanism underlying this reprogramming are undefined.

More recently, single cell RNA-seq and ATAC-seq analyses of human nasal polyp tissue uncovered an aberrant basal cell differentiation trajectory and demonstrated that basal progenitors retain an intrinsic memory of IL-4/IL-13 exposure *ex vivo* (48). Crucially, this memory persisted after removal from the inflammatory environment and was supported by sustained chromatin accessibility at inflammatory loci.

Although these studies establish a principle of inflammatory memory, reprogramming of terminally differentiated epithelial cells cannot provide durable memory to respiratory virus infections *in vivo*. For example, RSV and influenza infections stimulated terminally differentiated ciliated epithelial cells to undergo programmed apoptosis and sloughing (19, 22), so that memory in these populations would be lost.

### 4.2 Importance of memory in TP63^+^ basal cells

By contrast, basal cells represent the stem and progenitor compartment of the airway epithelium. Lung basal cells are long lived, self-renewing, and responsible for regenerating all major epithelial lineages (52). This positions basal cells, rather than mature epithelial cells, as an important reservoir for storing and transmitting inflammatory memory over time. In contrast to sloughing and apoptosis of terminally differentiated ciliated epithelial cells induced by RSV infections, the viral resistant population of the TP63+/KRT5+ population enters EMP, migrating to areas of injury giving rise to ciliated and secretory goblet cells to restore the epithelial barrier (19, 21).

Consequently, understanding the innate mechanisms providing viral resistance to the basal cell population is of intense interest. First described in pluripotent and multipotent stem cells, the intrinsic IIR is an IFN-autonomous pathway that represents a core IRF1-dependent subset of IFN-stimulable genes that provides constitutive, broad spectrum protection from a wide variety of viral agents (25). IFN-independent activation of IRF1, through changes in terminal differentiation enhances the basal tone of the intrinsic IIR, providing increased protection to viral attack. We suggest that basal cells modulate the intrinsic IIR tone so that prior exposed cells respond faster and more robustly to viral PAMPs.

### 4.3 IRF1 mediates intrinsic innate immunity and promotes memory

IRF1 is a ubiquitously expressed, highly conserved transcriptional factor known to play a central role in the initial triggering of the antiviral defense, inflammatory signaling, and immune epithelial communication (53). Animals or cells deficient in IRF1 have higher susceptibility to RNA viral infections in particular (54, 55). Recent attention has focused on the IFN- independent role of IRF1 controlling tonic anti-viral gene expression. Upon activation, IRF1 cross- and auto-regulates the family of IRFs inducing expression of IFNs, PRRs, potently upregulating the type I IFNs, notably IFNß (56), type III IFNs (38), and RIG I helicase (41) making dissection of the IFN-independent and IFN-dependent activities challenging. In this study, we have focused on *BST2, IFITM1* and *OAS2* genes as well-established markers of intrinsic IIR (24, 57). These genes are activated by IRF1 in cells lacking IFN or IFN receptors (53, 54), are directly bound IRF1 in unstimulated cells, and their regulation is rate-limited by ambient IRF1 levels (24).

Our earlier work showed that IRF1 functions as a pioneer transcription factor, maintaining enhancer function (53) coordinating activity of the BRD4 CRC and activated Ser2 phosphorylated RNA Pol II to innate responsive genes (24). Here, we find that repetitive IRF1 activation mediates hyper-responsiveness to TLR3 challenges priming. These events result in accumulation of EP300 on intrinsic IIR promoters without changes in IRF1 or BRD4 binding. Our data show that increased EP300 mediates inducible H3K27ac formation and enhanced innate responses. Further work will be required to examine whether these changes are coupled to stable epigenetic changes, such as chromatin opening.

### 4.4 BRD4 chromatin remodeling complex operates at the proximal promoters of innate genes in anti-viral responses

In the setting of innate activation, BRD4 CRC is dynamically recruited to proximal promoter regions that regulate genes involved in EMP and the IIR (32). This dynamic genomic redistribution is dynamically governed by interactions with acetylated transcription factors (58), the SMARCA4 chromatin remodeling complex (32) and acetylated histones (59). Our ChIP sequencing analysis demonstrates that BRD4 preferentially localizes to chromatin regions enriched in H3K27ac, consistent with earlier work (60).

The mechanisms how BRD4 regulates gene expression in proximal promoters has been extensively documented. Here, BRD4 functions as a core component of the positive transcription elongation factor b (P TEFb) complex, which contains cyclins and cyclin dependent kinase 9 (CDK9). Through this complex, BRD4 promotes phosphorylation of Ser2 within the carboxy terminal domain (CTD) of RNA Pol II and facilitates phosphorylation of negative elongation factors, thereby enabling productive transcriptional elongation and rapid induction of IIR and EMP (26).

Our findings extend this mechanism to indicate that BRD4 activity is modulated through its functional interaction with EP300 in response to repetitive TLR3 activation. Although we do not observe changes in BRD4 cellular abundance or increased BRD4 binding to IIR genes in trained cells, we find that the enhanced H3K27ac deposition characteristic of training is blocked by BRD4 inhibitor treatment. Because H3K27ac is deposited by EP300, this result indicates that BRD4 acts upstream of, or in concert with, EP300 to license this histone modification. Together, these findings suggest that training enhances BRD4-EP300 functional coupling, rather than enhancing BRD4 recruitment to innate promoters.

In this study, we have employed highly selective BRD4 bromodomain binding inhibitors with limited toxicity (61). We recognize that these BRD4 inhibitors may have unknown off-target effects and that our study would be enhanced by parallel BRD4 genetic depletion. However, we have not been able to develop BRD4 protein deficient hSAECs. These findings are consistent with those of others showing that cells compensate to CRISPR/Cas9-mediated BRD4 stable knockdowns bypassing the frameshift to preserve BRD4 expression (62).

### 4.5 Innate activation remodels the BRD4 CRC

The dynamic composition BRD4 CRC has been extensively investigated using unbiased proteomics, where we have found that RSV infection reconfigures the BRD4 complex to incorporate mediator complex, RNA Pol II, AP1 and RNA splicing factors to acutely activate innate response genes (44, 63). Here, our co-IP studies indicate that the BRD4 CRC also interacts with ubiquitous histone acetyltransferases which cooperate with BRD4 in mediating long-term innate memory. In the context of innate training, we conclude that the BRD4 CRC functionally interacts with EP300 to catalyze H3K27 acetylation. This conclusion is based on our findings that: 1). Training enhances EP300 to bind to the IIR genes; 2). pharmacologic inhibition of EP300 attenuates potentiated response of training and 3). EP300 inhibition reduces H3K27ac binding to the IIR.

Of particular mechanistic relevance, the BRD4 bromodomains interacts with both the COOH and NH_2_ terminal domains of EP300 (60), explaining the activity of the BD1 inhibitor on H3K27ac. *In vitro*, BRD4 enhances the histone acetyltransferase activity of EP300, resulting in increased H3K27 acetylation, indicating that this protein-protein interaction directly affects chromatin remodeling. Further investigation will be necessary to delineate the distinct contributions of the BRD4–EP300 complex to nucleosome repositioning and enhancer formation during the establishment of innate immune memory.

### 4.6 IRF1-EP300 interaction- an additional mechanism for EP300 activation

IRF1 plays an important coactivator role in type I IFN expression, mediating recruitment of EP300 coactivator to the PRDI-III domain of the IFNß enhanceosome (56). Other studies have shown that IRF1 also stimulates EP300 acetyltransferase activity (64) by the IRF1 COOH terminal transactivation domain binding the NH2 terminus of EP300 and stimulating its activity. Beyond the scope of this study, more work will be required to understand the multiple protein-protein interactions controlling EP300 histone acetylation in the setting of innate training.

### 4.7 Innate training couples to initiation of epithelial-mesenchymal plasticity

An important finding of our study is that IRF1 initiates EMP programs in trained epithelial cells, linking induction of the innate IIR with activation of cellular plasticity. EMP is a coordinated, step-wise series of partial cell-state changes that result in stable EMT (65). Here we extend the understanding of EMP initiation, linking repetitive TLR3 activation of the IRF1 transcription factor with EMP. Specifically, TLR3 activation drives upregulation of VIM, a cytoplasmic cytoskeletal protein responsible for enhancing cell motility. Additionally, we find IRF1 is required for upregulation of the mesenchymal genes, ZEB1 and MMP9, as well as the mesenchymal growth factor, IL6. In normal airways, basal cells are tightly adherent to laminins and type IV collagen within the basal lamina through cadherin-integrin interactions. With EMP, MMP9 secretion of MMP9 degrades the ECM, loosening this adherent interaction, while ZEB1 represses expression of epithelial cadherin, and VIM production promotes cytoplasmic motility. In this way, TLR3 mediated IRF1 activation initiates the EMP network to promote basal cell dissociation from its basal lamina to promote cytokinesis and regeneration of the epithelial barrier. In separate studies, the effects of memory in skin epithelial cells has been examined where TLR6 agonist (Imiquimod) enhances re-epithelialization of wounded skin (66). These data suggest that inflammation-memory is coordinated with EMP across many TLR receptor pathways.

## 5 Limitations

There are several limitations to this study. First, we focused on a single basal epithelial cell type. We recognize that multiple cell types in the intact airway may modify the acquisition and maintenance of innate memory. Extending this work to complex, multicellular tissue models will be an important next step. Second, we were unable to achieve stable genetic validation of BRD4 loss of function. In our hands, both shRNA- and CRISPR/Cas9-mediated BRD4 targeting in human small airway basal epithelial cells produced strong selective pressure and evidence of clonal adaptation, consistent with BRD4’s essential role in epithelial cell viability and transcriptional homeostasis. This precluded generation of a stable BRD4-null line and instead points to the need for inducible or acute depletion strategies (e.g., degron-based or transient knockdown approaches) to more cleanly isolate BRD4’s contribution without confounding by selective outgrowth.

## 6 Conclusions

In conclusion, we advance the mechanistic understanding of innate training in basal cells of the airway, linking the processes of intrinsic immunity with EMP. Our study suggests IRF1 functionally modifies BRD4-EP300 activity on proximal promoters of innate genes to enhance inducible histone acetylation. These interactions provide opportunities for translational modification of initial host response to virus and long-term phenotypic changes associated with repetitive viral infections.

## Data Availability

Publicly available BRD4 (GEO GSE170772) and EP300 ChIP-Seq data sets (ENCODE ENCSR571KWZ) from unstimulated type II-like A549 cells are available on the GEO.
